# A selected small molecule prevents inflammatory osteolysis through restraining osteoclastogenesis by modulating PTEN activity

**DOI:** 10.1002/ctm2.240

**Published:** 2020-12-01

**Authors:** Yueqi Chen, Wenhui Hu, Yiran Wang, Yuheng Li, Xiaoming Li, Haibo Li, Yong Tang, Lincheng Zhang, Yutong Dong, Xiaochao Yang, Ye Wei, Shiwu Dong

**Affiliations:** ^1^ Department of Biomedical Materials Science Third Military Medical University (Army Medical University) Chongqing PR China; ^2^ Department of Orthopaedics, Southwest Hospital Third Military Medical University (Army Medical University) Chongqing PR China; ^3^ National Engineering Research Center of Immunological Products & Department of Microbiology and Biochemical Pharmacy, College of Pharmacy Third Military Medical University (Army Medical University) Chongqing PR China; ^4^ School of Chemistry and Chemical Engineering Southwest University Chongqing PR China; ^5^ State Key Laboratory of Trauma, Burns and Combined Injury Third Military Medical University (Army Medical University) Chongqing PR China

**Keywords:** inflammatory osteolysis, osteoclastogenesis, PTEN, small molecule

## Abstract

**Background:**

Inflammatory osteolysis is a severe infectious bone disorder that occurs during orthopaedic surgery and is caused by disruptions in the dynamic balance of bone matrix homeostasis, which makes this condition a burden on surgical procedures. Developing novel therapeutic drugs about inhibiting excessive osteoclastogenesis acts as an efficient approach to preventing inflammatory bone destruction.

**Methods:**

To study this, we explored the potential effects and mechanisms of compound 17 on inflammatory osteolysis in vitro. Meanwhile, a lipopolysaccharide (LPS)‐induced calvarial osteolysis mouse model was used to evaluate the protective effect of compound 17 on inflammatory bone destruction in vivo.

**Results:**

In our study, we found that compound 17 could inhibit osteoclast (OC) differentiation and bone resorption during RANKL and LPS stimulation in a time‐ and dose‐dependent manner, while compounds 5 and 13 did not have the same effects. Mechanistically, compound 17 promoted phosphatase and tensin homologue (PTEN) activity by reducing PTEN ubiquitination, thereby restraining the RANKL‐induced NF‐κB pathway, resulting in the inhibition of the expression of osteoclastogenesis‐related genes and the formation of the NLRP3 inflammasome. Additionally, we also investigated whether compound 17 could negatively modulate macrophage polarization and repolarization due to its anti‐inflammatory effects. Moreover, compound 17 also plays an important role in osteoblast differentiation and mineralization. In vivo experiments showed that compound 17 could effectively protect mice from LPS‐induced inflammatory bone destruction by inhibiting osteoclastogenesis and inflammation.

**Conclusions:**

Taken together, these results show that compound 17 might play protective role in inflammatory bone destruction through inhibiting osteoclastogenesis and inflammation. These findings imply a possible role of compound 17 in inflammatory osteolysis‐related diseases.

## Graphical abstract

A selected compound 17 small molecule suppressed osteoclastogenesis in vitro by inducing PTEN activity by restraining PTEN ubiquitination, thus inhibiting the RANKL‐induced NF‐κB pathway. The polarization and repolarization of M1 macrophages were also negatively modulated. In vivo experiments showed that compound 17 prevented LPS‐induced inflammatory osteolysis by inhibiting OC activity. All in all, compound 17 might act as a potential novel drug to treat inflammatory osteolysis‐related diseases in the future.

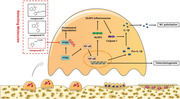

**Abbreviations**
ALP: alkaline phosphatase
Arg‐1: arginine synthase‐1
ARS: alizarin red S
ASC: apoptosis‐associated speck‐like protein containing a CARD
BMD: bone mineral density
BMMs: bone marrow macrophages
BV/TV: bone volume/tissue volume
CCK‐8: cell counting kit‐8
c‐Fos: cellular protooncogene Fos
CTSK: cathepsin k
DAPI: 4′,6‐diamidino‐2‐phenylindole
DC‐STAMP: dendritic cell‐specific transmembrane protein
DMSO: dimethyl sulfoxide
F‐actin: fibrous actin
FITC: fluorescein isothiocyanate
IFN‐γ: interferon‐γ
IL: interleukin
iNOS: inducible nitric oxide synthetase
ITAM: immunoreceptor tyrosine‐based activated motif
LPS: lipopolysaccharides
M‐CSF: macrophage colony‐stimulating factor
MMP: matrix metalloproteinases
NFATc1: nuclear factor of activated T cell c1
NF‐κB: nuclear factor kappa‐light‐chain‐enhancer of activated B cells
NLRP3: NOD‐like receptor family pyrin domain containing 3
OBs: osteoblasts
OCs: osteoclasts
OM: osteomyelitis
OSCAR: osteoclast‐associated immunoglobulin‐like receptor
PTEN: phosphatase and tensin homolog deleted on chromosome ten
qPCR: quantitative real‐time polymerase chain reaction
RANKL: receptor activator of NF‐κB ligand
Tb.N: trabecular number
Tb.Sp: trabecular separation
Tb.Th: trabecular thickness
TNF‐α: tumour necrosis factor α
TRAP: tartrate‐resistant acid phosphatase
μCT: micro‐computed tomography

## 1 INTRODUCTION

Bone acts as a rigid tissue and undergoes continuous remodelling to regulate bone matrix homeostasis and maintain the dynamic balance of the microenvironment in the bone marrow cavity.^1^ Bone remodelling is a complex biological process that involves osteoblast‐mediated bone formation and osteoclast (OC)‐mediated bone resorption.^2^ Pathological bone loss often occurs due to excessive OC formation and bone resorption, resulting in the poor progression of many severe sterile and non‐sterile inflammatory osteolysis‐related diseases. Sterile inflammatory osteolysis, for example, is caused by wear debris around a prosthesis, while septic osteolysis leads to infectious diseases such as osteomyelitis (OM).^3^ With the gradually increasing number of bone infection clinical trials around the world, therapeutic strategies are focused on anti‐infection and anti‐osteolysis, which have been recognised as strong economic burdens. Therefore, developing effective prevention and treatment strategies have attracted more attention.

OM is the most challenging inflammatory degenerative bone disease in clinical medicine.^4^ These inflammatory processes include pathological destruction and necrosis of the local bone tissue, sequestrum formation and apposition of new bone tissue.^5^, ^6^ Lipopolysaccharide (LPS), which is a component of the outer membrane of all gram‐negative bacteria, was one of the first bacterial components to be widely implicated in the process of bone resorption during the progression of infection, and LPS can also induce the formation of the NLRP3 inflammasome and trigger the release of many pro‐inflammatory cytokines by macrophages, such as interleukin‐1β (IL‐1β), IL‐6 and tumour necrosis factor‐α (TNF‐α), to subsequently initiate inflammatory cascades that result in severe inflammatory bone destruction.^7^ Moreover, it has been suggested that LPS could also contribute to the formation of the NLRP3 inflammasome during bone infection.^8^ In addition, LPS has been shown to act as an inducer in the rapid and robust formation of OCs, as well as the expansion of eroded bone areas.^8^

Haematopoietic monocyte/macrophage‐like stem cells have been reported to form mature OCs after stimulation with macrophage colony stimulating factor (M‐CSF) and receptor activator of nuclear factor kappa‐B ligand (RANKL) during osteoclastogenesis.^9^ The process of OC differentiation consists of several different stages, such as the differentiation of tartrate‐resistant acid phosphatase (TRAP)‐positive cells, fusion into multinucleated cells, the initiation of bone erosion and the induction of spontaneous apoptosis.^10^ After RANKL binds to its receptor, RANK directly activates a series of intracellular signalling pathways, including the nuclear factor kappa‐light‐chain‐enhancer of activated B cells (NF‐κB) and phosphatase and tensin homologue (PTEN) pathways, subsequently stimulating the expression of OC differentiation‐related downstream factors, such as nuclear factor of activated T cells 1 (NFATc1).^11^ NFATc1 has been reported to be a master regulator that modulates the expression of OC differentiation‐related genes, including TRAP, cathepsin K (CTSK), matrix metalloproteinase‐9 (MMP‐9) and others.^12^

The ubiquitin system has been reported to play an essential role in a large number of processes in the bone microenvironment and provides attractive drug targets for the treatment of skeletal system diseases.^13^ Homologous to E6AP C terminus (HECT) domain ligases receive ubiquitin from an E2 enzyme and transfer it to the substrate.^14^ Inhibitors of domain ligases demonstrate that HECT domains are the targets of drugs and protect against bone loss.^15^ It has been reported that the small molecule heclin (an HECT ligase inhibitor) showed inhibitory effects on several HECT ligases in tissue culture by contributing to the oxidation of the active site of cysteine.^16^ Heclin also exhibits the obvious basic structure of the Michael acceptor system, which also belongs to compound of acrylamide.^16^ Interestingly, Michael acceptor system molecules can regulate numerous signal transduction pathways or enzymatic activities in cells through covalent binding with cysteine or arginine residues and then initiate the Michael addition reaction to exert anti‐inflammatory or antioxidant functions.^17^ Thus, we synthesized many compounds based on the structure of heclin and compared their potential biological activities.^16^, ^18^

**STUDY HIGHLIGHTS**

1. **What is the current knowledge on the topic?**
  Bone infection resulting in severe inflammatory osteolysis, which is common in orthopaedic surgery. Excessive osteolysis caused by the enhanced OC fusion and differentiation during inflammatory condition. Many therapeutic approaches focused on the anti‐inflammation and the deletion of infectious bone tissue. However, it is also necessary to develop novel drugs to restrain excessive inflammatory bone destruction.
2. **What question did this study address?**
  We performed this study to make the further investigation about effects and potential mechanisms of the small molecule on inflammatory osteolysis in vitro and in vivo.
3. **What does this study add to our knowledge?**
  Our results showed that compound 17 inhibited RANKL‐induced osteoclastogenesis through PTEN dependent NF‐κB signaling pathway, and restrained the expression of osteoclastogenesis‐related genes and the formation of the NLRP3 inflammasome. Meantime, the macrophages polarization and repolarization, the release of inflammatory cytokines could also be negatively regulated. Furthermore, the animal model of LPS‐induced inflammatory bone destruction could also be protected after treatment with compound 17.
4. **How might this change clinical pharmacology or translational science?**
  This small molecule named as compound 17 may be a potential therapeutic agent for inflammatory bone destruction.

In the present study, we performed further investigation by selecting a small molecule (compound 17) that acts as the most similar inhibitor of the HECT domain and could inhibit RANKL‐induced osteoclastogenesis by attenuating NF‐κB signalling and the formation of the NLRP3 inflammasome by regulating the ubiquitination of PTEN. We also evaluated the protective effects of compound 17 on inflammatory bone loss upon LPS‐induced calvarial inflammatory osteolysis in vivo.

## 2 MATERIALS AND METHODS

### 2.1 Reagents and antibodies

Dimethyl sulfoxide (DMSO) was purchased from Sigma‐Aldrich (St. Louis, MO). Recombinant mouse M‐CSF and mouse RANKL were purchased from R&D Systems (Minneapolis, MN). Osteo Assay Surface for bone resorption assay was purchased from Corning (NY). Bovine cortical bone slices for pit formation assay were obtained from Boineslices.com (Jelling, Denmark). TRAP stain kit and LPS from *Escherichia coli* O55:B5 were obtained from Sigma‐Aldrich (NY). Actin Cytoskeleton and Focal Adhesion Staining Kit was purchased from Millipore (Darmstadt, Germany). All cell culture reagents were purchased from Thermo Fisher Scientific Inc. (Gibco, Waltham, MA). Penicillin‐streptomycin solution was obtained from Hyclone (Thermo Scientific). Mouse IL‐1β, IL‐6 and TNF‐α ELISA Kit were obtained from Dakewe Biotech Co. Ltd (Shenzhen, China). Recombinant murine IL‐4, IL‐13 and interferon (IFN)‐γ protein were obtained from PeproTech Inc (Rocky Hill, NJ). Antibodies against alkaline phosphatase (ALP), MMP9, CD9, TRAP, CTSK, c‐Fos and NFATc1 were obtained from Cell Signaling Technology Inc (Danvers, MA). Antibodies against BMP‐2, phospho‐NFκB p65, NF‐κB p65, phospho‐IκBα, IκBα, PTEN and β‐actin were obtained from Bioworld Technology (St. Louis Park, MN). Antibodies against caspase‐1, NLRP3, IL‐1β, apoptosis‐associated speck‐like protein containing a CARD (ASC), Arg‐1 and inducible nitric oxide synthetase (iNOS) were obtained from Biosynthesis Biotechnology Inc. (Beijing, China). Antibody against ubiquitin was purchased from Abcam (Cambridge, United Kingdom). MC3T3‐E1 cells were obtained from the American Type Culture Collection (Rockville, MD). VO‐Ohpic, MG132 and cycloheximide (CHX) were purchased from MedChemExpress (Monmouth Junction, NJ). All compounds were dissolved in DMSO and stored at −20°C. Then compounds were diluted in cell culture medium so that DMSO comprised <0.1% of the total volume during the experiments.

### 2.2 Preparation, characterisation and selection of small molecules

The formulas of all the compounds (no.1 to no.17) are shown in the Figure S5. The detailed process of choosing the small molecules is based on the basic structure of the small molecule heclin (an HECT ligase inhibitor). We found that compounds 5, 13 and 17 seem like performing the high similarity when comparing with heclin. Then the detailed synthesis and characterisation of compounds 5, 13 and 17 were also elucidated in supporting information.

### 2.3 Cell culture and cell viability assay

Mouse primary bone marrow monocytes/macrophages (BMMs) were harvested from the femur and tibiae of thirty C57BL/6J mice (female, 6‐8 weeks, Beijing Huafukang Bioscience Co. Inc.). They were aseptically removed, and the bone marrow cells were flushed out. The cells were suspended and cultured in α‐minimum Eagle's medium (α‐MEM, Gibco) with 10% of fetal bovine serum (FBS) (Gibco), 1% of penicillin‐streptomycin solution (Gibco) and 30 ng/mL of mouse M‐CSF (R&D) overnight at 37°C. The untouched cells were collected and further cultured with 50 ng/mL M‐CSF for 2 days to obtain BMMs.

BMMs were seeded (2 × 10^3^/well) into 96‐well plates and were cultured overnight. Then they were induced with M‐CSF (50 ng/mL) and RANKL (100 ng/mL) for 24 hours or 72 hours with different dosages of compounds 5, 13 and 17. Cytotoxicity assay was evaluated by Cell Counting Kit‐8 (Dojindo, Japan) reagent at 24 hours, and 72 hours according to the manufacturer's instructions. The absorbency of cells was measured using a 96‐well plate reader at 450 nm. Wells containing the CCK‐8 reagent with no cells were defined as the blank control. Absorbance at 450 nm was evaluated by using multi‐detection microplate reader (BioTek Instruments, Winooski, VT) according to the manufacturer's instructions.

### 2.4 In vitro assays for OC differentiation, fusion and bone resorption activity

BMMs were cultured with RANKL (100 ng/mL) and M‐CSF (50 ng/mL) to obtain pre‐OCs. Then the cells were incubated with RANKL (100 ng/mL) and M‐CSF (50 ng/mL) or LPS for several days to obtain mature OCs. In order to evaluate the effects of compound 17 on the several stages of OCs during osteoclastogenesis, we have added the compound 17 into these early stage and mid late stage.

To observe the effect of compound 17 on OC differentiation, TRAP stain was performed. Cells were fixed in 4% paraformaldehyde for 20 minutes and then stained with TRAP staining solution (0.1 mg/mL of naphthol AS‐MX phosphate, 0.3 mg/mL of Fast Red Violet LB stain) according to the manufacturer's instructions. TRAP‐positive multinucleated (nuclei >3) cells were counted as OCs using a light microscope (DMI 6000B; Leica Microsystems, Wetzlar, Germany).

To verify its role in the OC fusion, actin cytoskeleton and focal adhesion (FAK) stain was completed. Mature OCs were washed and fixed for permeabilization. After blocking, primary antibody (anti‐vinculin) was then diluted to a working concentration (1:300) in blocking solution, and cells were incubated for 1 hour at room temperature. Secondary antibody (Alexa Fluor 488 Goat Anti‐Mouse IgG [H+L] Antibody, Invitrogen) (1: 500) and TRITC conjugated phalloidin (1: 500) were diluted in 1 × PBS (Phosphate Buffered Saline), and cells were incubated for 1 hour at room temperature. Nuclei staining was performed by 4ʹ, 6‐diamidino‐2‐phenylindole (DAPI) (1:1000) for 5 minutes followed by confocal laser scanning microscopy (Nikon Ti‐E imaging system, Tokyo, Japan). Many detailed procedures were described in previous study.^19^

To investigate the change of mature OCs mediated bone resorption activity, pit formation assay was greatly performed. OCs were incubated onto 96‐well plates Osteo Assay Surface and bovine bone slices. After observing the multinucleated mature OCs at light microscopy, 0.1 % methylene blue stain was performed to evaluate the resorption area on bone slices. Bleach solution was added to 96‐well osteo surface plates to remove cells. Detailed analysis of pit formation area was described previously.^20^

### 2.5 Flow cytometric analysis

To analyse the effect of compound 17 on cell apoptosis during osteoclastogenesis, BMMs were induced with RANKL (100 ng/mL) and M‐CSF (50 ng/mL) for 72 hours with treatments of compound 17 at different dosages (0, 5μM, 10μM, 25μM, 50μM). Cells were washed twice with cold PBS and then resuspended in 500 μl of binding buffer (10 mM HEPES/NaOH [pH 7.4], 140 mM NaCl, 2.5 mM CaCl2) at a concentration of 1 × 10^6^ cells/mL. Cells were then stained with 5 μl of annexin V‐FITC (Life Technologies) and 10 μl of 20 μg/mL PI. Apoptosis was analysed using a FACStar flow cytometer (BD).

### 2.6 Nuclear translocation fluorescence staining

BMMs cells were incubated in the presence or absence of 10μM compound 17 for 1 hour before treatment with RANKL for an additional 60 minutes. Cells were fixed with 4% paraformaldehyde for 20 minutes. Then cells were permeabilized and blocked before the cells were incubated with the primary antibody (NF‐κB p65, 1:200) at 4°C. Cells were washed three times with PBS and then incubated with an Alexa 488 nm‐conjugated secondary antibody and DAPI in the dark. The intensity value was analysed by using the Image J software (NIH, Bethesda, MD).

### 2.7 Osteoblast differentiation, ALP and alizarin red S staining

Clonal osteoblastic MC3T3‐E1 cells were cultured in α‐MEM with 10% FBS, 20 mM HEPES and 1% penicillin‐streptomycin. To detect the differentiation of osteoblast, MC3T3‐E1 cells were seeded (2 × 10^4^ cells/cm^2^) in 12‐well dishes and cultured in osteogenic medium (1 mM β‐glycerophosphate, and 5μM L‐ascorbic acid 2‐phosphate) with different concentrations of compound 17 (0, 10 and 25μM).

To evaluate the effect of compound 17 on osteoblastogenesis, MC3T3‐E1 cells were also seeded and incubated as the same methods for 7 days. Then cells were washed with PBS twice and fixed with 4% paraformaldehyde for 30 minutes at room temperature. A BCIP/NBT staining kit (C3206, Beyotime Institute of Biotechnology, Shanghai, China) was used after cell fixation.

To investigate the effect of compound 17 on bone matrix mineralization, MC3T3‐E1 cells were also seeded and incubated as the same methods. After cells were grown in a differentiation‐inducing medium for 14 days, washed three times with ddH2O, and then fixed in 95% alcohol for 15 minutes. The cells were then incubated in 1% alizarin red S (ARS) (Sigma‐Aldrich) solution for 15 minutes and washed three times with ddH2O at room temperature before imaging under a microscope.

### 2.8 In vitro modulation of macrophage polarization and repolarization

Many other related reports have noted that M1‐MΦ and M2‐MΦ participated in the inflammatory osteolysis.^21^, ^22^ In order to obtain M1‐MΦ, BMMs were stimulated with 50 ng/mL LPS or 20 ng/mL IFN‐γ for another 24 hours. Individually, to generate M2‐MΦ, the BMM cells were incubated with 20 ng/mL IL‐4 or 20 ng/mL IL‐13 in the presence of 30 ng/mL M‐CSF for 24 hours. Respectively, after washing, the M1‐MΦ were further treated with 20 ng/mL IL‐4 or 20 ng/mL IL‐13 for another 24 hours to perform the procedure of repolarization from M1‐MΦ to M2‐MΦ. To evaluate the effect of compound 17 on the macrophage polarization and repolarization, we have added this compound 17 during the M0‐MΦ to M1‐MΦ and M0‐MΦ to M2‐MΦ.

### 2.9 RNA extraction and quantitative real‐time‐polymerase chain reaction

Quantitative real‐time polymerase chain reaction (qPCR) was utilized to evaluate gene expression during osteoclastogenesis, osteoblastogenesis and macrophage polarization. BMMs and MC3T3‐E1 cells were incubated with the same strategies as before. Total RNA was isolated using Trizol reagent (Life Technologies). Single‐stranded cDNA was prepared from 1 μg of total RNA using reverse transcriptase with oligo‐dT primer according the manufacturer's instructions (Promega, Madison, WI). Two micro‐litres of each cDNA were subjected to PCR amplification using specific primers shown in Table S1.

### 2.10 Western blot

BMMs and MC3T3‐E1 cells were incubated with the same strategies as before. Untreated cells served as a negative control group. For completing western blot analysis, total protein was extracted from the cultured cells using radioimmunoprecipitation assay lysis buffer (Sigma Aldrich). Cells were lysed in radio immunoprecipitation assay buffer for 30 minutes at 4°C and then the supernatants were collected. Proteins were separated by 10% sodium dodecyl sulfate polyacrylamide gel electrophoresis and transferred to polyvinylidene difluoride membranes (Bio‐Rad, Hercules, CA). The membranes were blocked in 5% non‐fat dry milk in Tris Buffered Saline Tween (50 mM Tris, pH 7.6; 150 mM NaCl; 0.1% Tween‐20) at room temperature for 1 hour and incubated with the primary antibodies overnight at 4°C followed by 1 hour incubation with secondary antibody. Enhanced chemiluminescence (Sigma‐Aldrich, St. Louis, MO) was used to visualise protein bands, and the relative grey level was analysed using Image J software.

### 2.11 PTEN immunoprecipitation and ubiquitination assay

To investigate the interaction between ubiquitin protein and PTEN protein, Co‐IP analysis was greatly performed. BMMs were treated with 10μM compound 17 for 12 hours in the presence of absence of 50μM MG132 for 2 hours, and then treated with 100 ng/mL RANKL and 50 ng/mL M‐CSF for 30 minutes. Cells were lysed in RIPA buffer (Thermo Fisher Scientific) after washes with PBS. The supernatant was incubated with anti‐PTEN antibody (1:100) or mouse IgG overnight at 4°C, followed by an incubation with Protein A/G Agarose (Thermo Fisher Scientific) for 4 hours at 4°C. The following day, IP of NF‐κB p65 was performed using Protein A/G Magnetic Beads (Thermo Fisher Scientific) according to the manufacturer's protocol. The complexes were washed with PBS and subjected to western blot analysis using antibodies against ubiquitin (1:1000). Blots against β‐actin served as loading control.

### 2.12 LPS‐induced inflammatory bone destruction model

All procedures involving mice and experimental protocols were approved by Institutional Animal Care and Use Committee of Third Military Medical University. Mice for all experiments were females 6‐8 weeks of age, which were supplied by the Animal Experiment Center of Third Military Medical University. We constructed LPS‐induced inflammatory bone destruction murine model in mouse calvaria based on our previous study.^8^ Fifteen C57BL/6J mice (female, 20‐22 g) were randomly divided into three groups (n = 5): (a) PBS control, (b) LPS (10 mg/kg body weight) and (c) LPS (10 mg/kg body weight) + compound 17 (5 mg/kg body weight). All animals were anaesthetized after injecting with 4% chloral hydrate (5 mL/kg) intraperitoneally to reduce the level of suffering. The heads of the anaesthetized mice were shaved to receive subperiosteal injections locally and systemically. The total volume of the injections was 100 mL each time. After weighing the mice, the injections were administered with the 1 mL syringe at a point on the sagittal midline suture of the calvarium located between the ears and eyes. Next, a skin mound was formed, and then the needle was slowly withdrawn to prevent the liquid from overflowing. Subsequently, the injections were administered every 2 days for 4 weeks. Following the final injection, mice were sacrificed by cervical dislocation, and their calvaria and femur were collected and fixed in 4% paraformaldehyde for 2 days.

### 2.13 Micro‐CT scanning analysis

Bruker micro‐CT Skyscan 1272 system (Kontich, Belgium) with an isotropic voxel size of 8.0μm was used to image the whole calvaria, while the isotropic voxel size of 5.0 μm was used for femur. Scans were conducted in 4% paraformaldehyde and used an X‐ray tube potential of 60 kV, an X‐ray intensity of 166 μA, and an exposure time of 1700 milliseconds. Three‐dimensional (3D) image reconstructions were taken, and SkyScan software was used for quantitative analysis. Regions of interest of equal area (3 mm in diameter) were identified in the centre of each calvaria. For trabecular bone analysis of the distal femur, an upper 3‐mm region beginning 0.8 mm proximal to the most proximal central epiphysis of the femur was contoured. For cortical bone analysis of femur (2D analysis), a 0.5‐mm region beginning 4.5 mm proximal to the most proximal central epiphysis of the femur was contoured. The threshold for the calvaria and femur were set at 86‐255 (8‐bit gray scale bitmap). μCT scans of whole body of mice (except skull) were performed using isotropic voxel sizes of 148 μm. Reconstruction was accomplished by Nrecon (Ver. 1.6.10). 3D images were obtained from contoured 2D images by methods based on distance transformation of the grey scale original images (CTvox, Ver. 3.0.0). All images presented are representative of the respective groups. SkyScan software was used to analyse bone mineral density (BMD), bone volume/tissue volume (BV/TV), trabecular number (Tb.N), trabecular thickness (Tb.Th) and trabecular separation (Tb.Sp).

### 2.14 Histological analysis

For histological analysis, after decalcification in 10% ethylene diamine tetraacetic acid (Sigma‐Aldrich, St. Louis, MO) for 14 days, the calvaria (n = 5/group) was embedded in molten paraffin. The tissues were cut into 6 μm sections and prepared for haematoxylin and eosin (H&E) and Masson's trichrome following the manufacturer's protocols. Images were captured using an AxioCam HRc microscope (Carl Zeiss, Germany). The bone eroded surface area ratio and bone thickness at the closest side of biparietal suture were determined using Image J. Blue‐colored new bone collagen fibre was observed using Masson's trichrome staining.

For immunohistochemical analysis, expression of TRAP, IL‐1β, NLRP3, CD206 and iNOS (inducible nitric oxide synthetase) in the calvaria was detected by immunohistochemistry (IHC) using the primary antibodies at the concentration of 1:100. In brief, the sections were dewaxed with xylene and then subjected to gradient hydration and antigen retrieval with hyaluronidase for 1 hour at 37°C and pepsin for 25 minutes at room temperature. The sections were then blocked with second antibody homologous serum for 30 minutes. Next, the paraffin sections were incubated with primary antibodies at 4°C. After 12 hours, the sections were rinsed with PBS and incubated with secondary antibodies for 30 minutes at room temperature. A DAB Horseradish Peroxidase Color Development Kit (Beyotime, Shanghai, China) was used to induce the chromogenic reaction. IHC‐positive cells, the ratio of OC surface‐to‐bone perimeter (Oc.S/OB) and the number of OCs to bone surface (N.Oc/BS) were determined using Bioquant Osteo 2017 (BIOQUANT Image Analysis Corporation, Nashville, TN).

### 2.15 Measurement of inflammatory cytokines

To detect the release of inflammatory cytokines, serum IL‐6, IL‐1β and TNF‐α levels were detected using a mouse IL‐6, IL‐1β and TNF‐α ELISA kit according to the manufacturer's protocol. The serum of mice was obtained from whole blood after standing for 30 minutes at room temperature and centrifugation at 3000 rpm for 10 minutes at 4°C. The absorbency of each standard and sample was measured at 450 nm. Standard concentration gradient was used as a standard curve.

### 2.16 Statistical analysis

All data are representative of at least three experiments of similar results performed in triplicate unless otherwise indicated. Data are expressed as mean ± SD. Statistical analysis was performed using SPSS ver. 19.0 software (SPSS, Chicago, IL). One‐way ANOVA followed by Student‐Newman‐Keuls post hoc tests was used to determine the significance of difference between results, with *P < .05, **P < .01 and ***P < .001 being regarded as significant. N.S. represented as the no significant difference.

## 3 RESULTS

### 3.1 The synthesis and characterisation of small molecules

**FIGURE 1:**
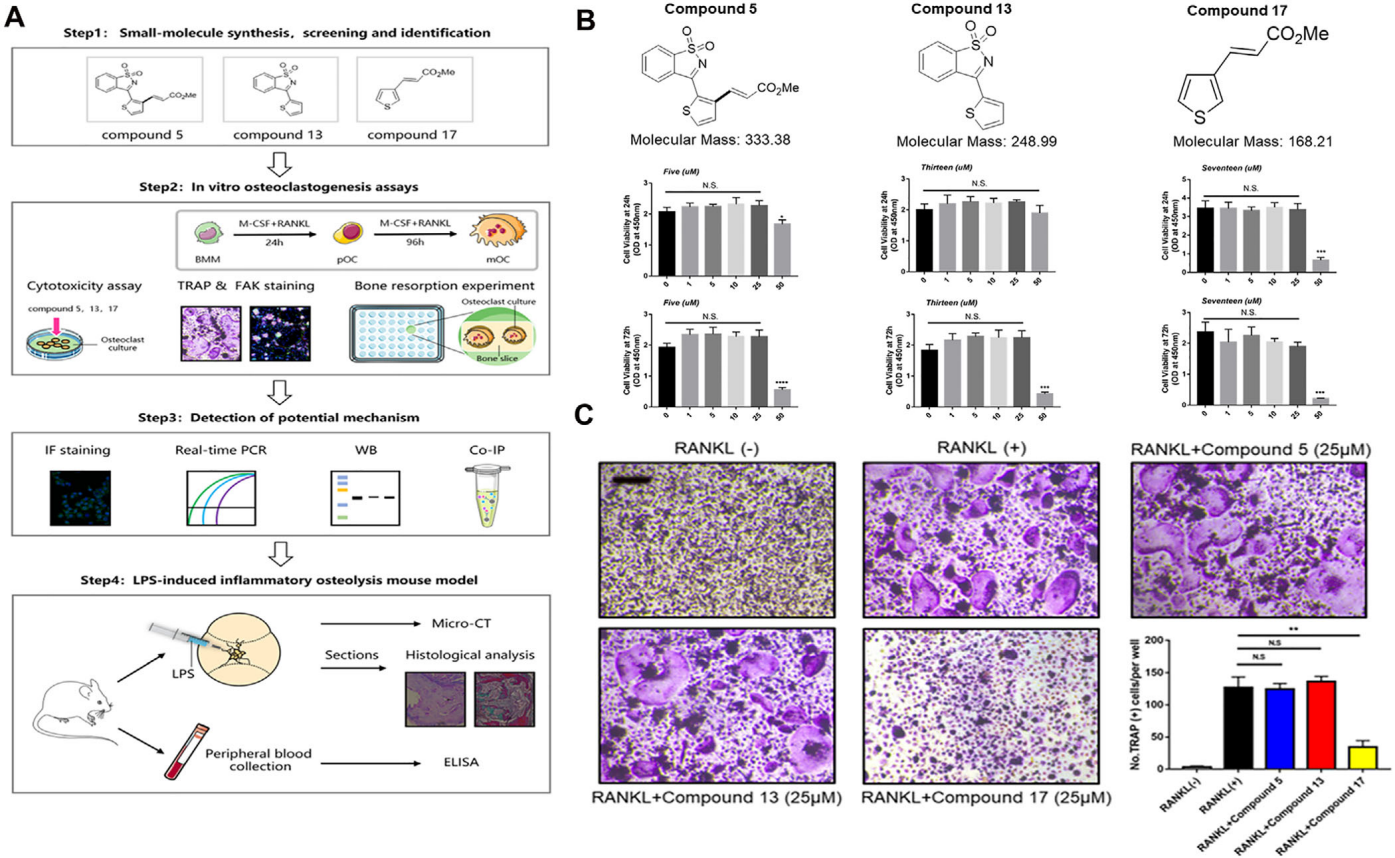
**Selection and evaluation about the effects of small molecules on osteoclasts differentiation**. A, Schematic diagram for design of evaluation about the effects on inflammatory osteolysis in vitro and in vivo. B, The chemical molecular formulas of these compounds and the cytotoxicity evaluation about them in the presence of RANKL and M‐CSF for 24 hours and 72 hours. C, Representative images of TRAP staining of BMMs cells cultured with different compounds in the presence of RANKL and M‐CSF for 6 days. Bar represents 200 μm. Images are representative of n = 3 independent experiments. The data in the figures represent the averages ± SD. Significant differences are indicated as * (*P* < .05), ** (*P* < .01) and *** (*P* < .001) paired using Student's *t*‐test unless otherwise specified. N.S. represented as the no significant difference

The synthetic compounds were completed as the follow procedures. This experimental design is shown in Figure 1A. The detailed chemical synthesis procedure and the nuclear magnetic hydrogen and nuclear magnetic carbon spectra are shown in the Figures S1‐S4.

### 3.2 The evaluation of compounds 5, 13 and 17 on cytotoxicity analysis and OC differentiation

To evaluate the potential cytotoxicity of these compounds, a CCK‐8 cell viability assay was performed. After incubation with different doses of compounds 5, 13 and 17 in the presence of RANKL and M‐CSF for 24 hours or 72 hours, cytotoxicity analysis showed that compounds 5 and 17 were not cytotoxic at concentrations less than 25 μM for either 24 hours or 72 hours. While compound 13 may be safe at doses less than 50 μM for 24 hours, there was potential cytotoxicity at a concentration of 50 μM for 48 hours (Figure 1B). In addition, we evaluated the potential role of these compounds in osteoclastogenesis. We added these compounds during OC differentiation and formation at a concentration of 25 μM for 6 days, and the results showed that compound 17 could reduce the number of TRAP‐positive cells, while compounds 5 and 13 exhibited no effective inhibitory functions, which suggested that compound 17 could play a suppressive role in osteoclastogenesis (Figure 1C). Additionally, cytotoxicity assays and flow cytometry were performed to verify whether the inhibitory effect of osteoclastogenesis was due to cytotoxic activity. The results indicated that concentrations of compound 17 ranging from 1 μM to 25 μM were not cytotoxic to BMMs (Figure 1B). Interestingly, we also found that 25 μM compound 17 could also induce the apoptosis of mature OCs during osteoclastogenesis (Figure S6). Thus, the suppressive effects of compound 17 on osteoclastogenesis were shown without potential cytotoxicity.

### 3.3 Compound 17 inhibited RANKL‐induced osteoclastogenesis in vitro

**FIGURE 2:**
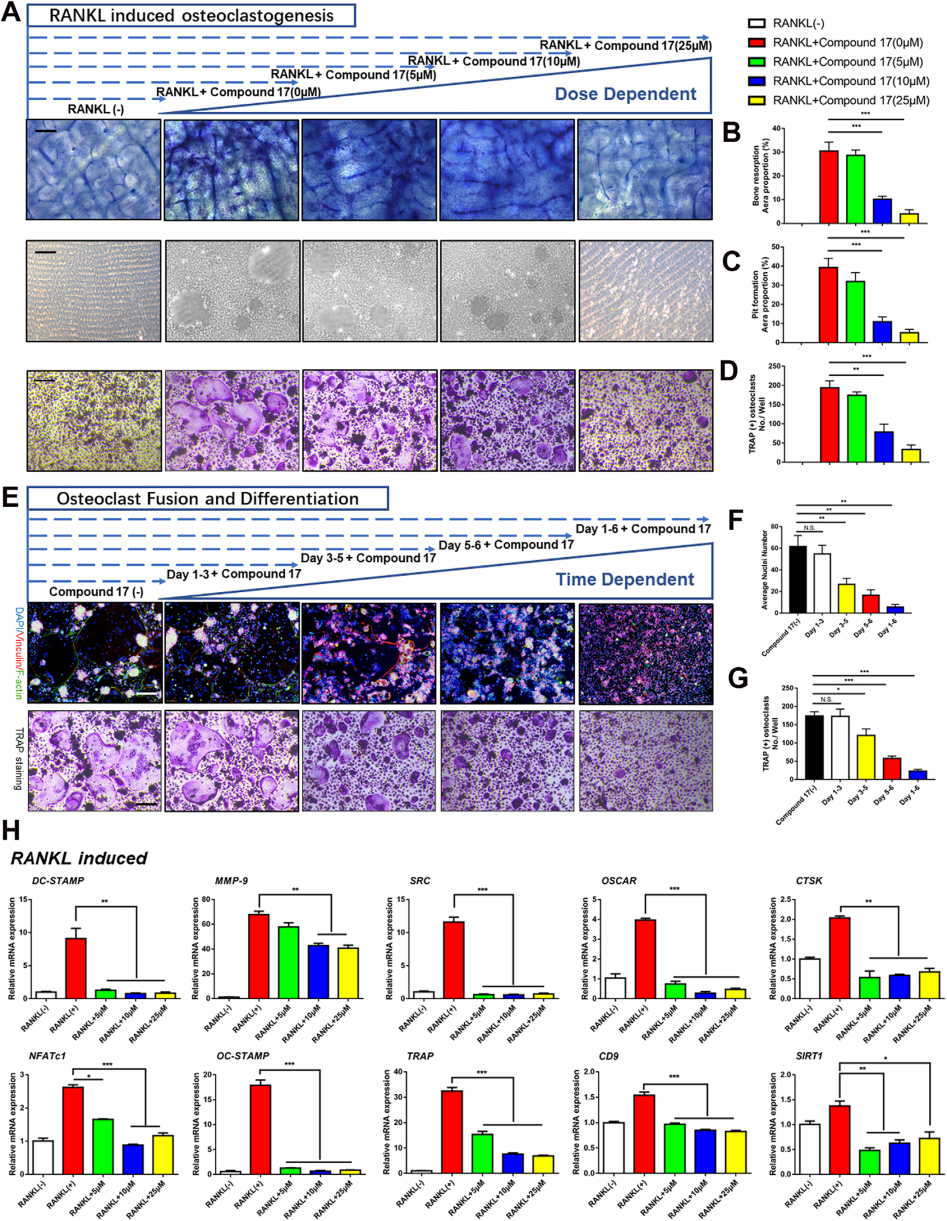
**Compound 17 inhibits RANKL‐induced osteoclast formation and bone resorption activity both in time and dose dependent manner**. A, Representative images of TRAP staining, bone resorption assay and pit formation assay of RANKL induced osteoclasts in the absence or presence with different concentration of compound 17 for 6 days. Bar represents 200 μm. Images are representative of n = 3 independent experiments. B, Quantification analysis of the proportion about bone resorption area. C, Quantification analysis of the proportion about pit formation area. D, Quantification analysis of the TRAP positive osteoclasts in each well. E, Representative images of TRAP staining and FAK staining of RANKL‐induced osteoclasts in the absence or presence with different concentration of compound 17 for various days. Bar represents 200 μm. Images are representative of n = 3 independent experiments. F, Quantification analysis of the average nuclei number in each osteoclast. G, Quantification analysis of the TRAP positive osteoclasts in each well. H, Relative expression of marker genes in the procedure of RANKL‐induced osteoclastogenesis from monocytes to mature osteoclasts at mRNA level. The data in the figures represent the averages ± SD. Significant differences are indicated as * (*P* < .05), ** (*P* < .01) and *** (*P* < .001) paired using Student's *t*‐test unless otherwise specified. N.S. represented as the no significant difference

To investigate the effects of compound 17 on OC differentiation and function, BMMs were induced to become OCs by RANKL in the presence of different concentrations of compound 17 for 6 days. The results showed that the number of TRAP‐positive OCs was suppressed compared to that of the positive control group in the absence of compound 17 treatment (Figures 2A and 2D). Increased doses of compound 17 inhibited TRAP‐positive OC formation (Figures 2A and 2D).

Moreover, the effects of compound 17 on the bone resorption activity of mature OCs were further examined by bone resorptive assays. BMMs were incubated with RANKL to form multinucleated mature OCs, after which an equal number of cells were seeded onto osteo assay surface plates and bovine cortical bone slices. Compound 17 significantly reduced the percentage of the resorbed area during OC differentiation and formation (Figure 2A‐C). These findings revealed that compound 17 could inhibit OC‐related bone resorption in a dose‐dependent manner. Moreover, to examine which stage of osteoclastogenesis was affected, BMM‐derived OCs were seeded with RANKL in the presence of 10 μM compound 17 for the indicated times. Compound 17 was found to predominantly exert its suppressive effect during the mid‐late stage (days 3‐6) of OC differentiation rather than the early stage (days 1‐3), which indicated the exact stages of osteoclastogenesis that were restrained after treatment with compound 17 (Figures 2E and 2G). Additionally, immunofluorescence analysis showed that compound 17 impaired F‐actin ring formation, as demonstrated by the decreased sizes of F‐actin rings and decreased average number of nuclei in OCs in the compound 17‐treated groups (Figures 2E,F).

At the molecular level, the expression of several OC‐specific genes, including NFATc1, DC‐STAMP, MMP‐9, OC‐STAMP, SRC, TRAP, OSCAR, CD9, CTSK and SIRT1, was upregulated in BMMs when mature OC differentiation was induced.^23^, ^24^, ^25^, ^26^, ^27^ We examined these genes at the mRNA level by using quantitative PCR to observe how compound 17 affected OC‐specific gene expression and found that the expression of these genes was inhibited following the administration of compound 17 (Figure 2H). Collectively, these results further verified that compound 17 suppressed osteoclastogenesis in both a time‐ and dose‐dependent manner.

### 3.4 Compound 17 attenuated LPS‐induced OC differentiation and bone resorption activity

**FIGURE 3:**
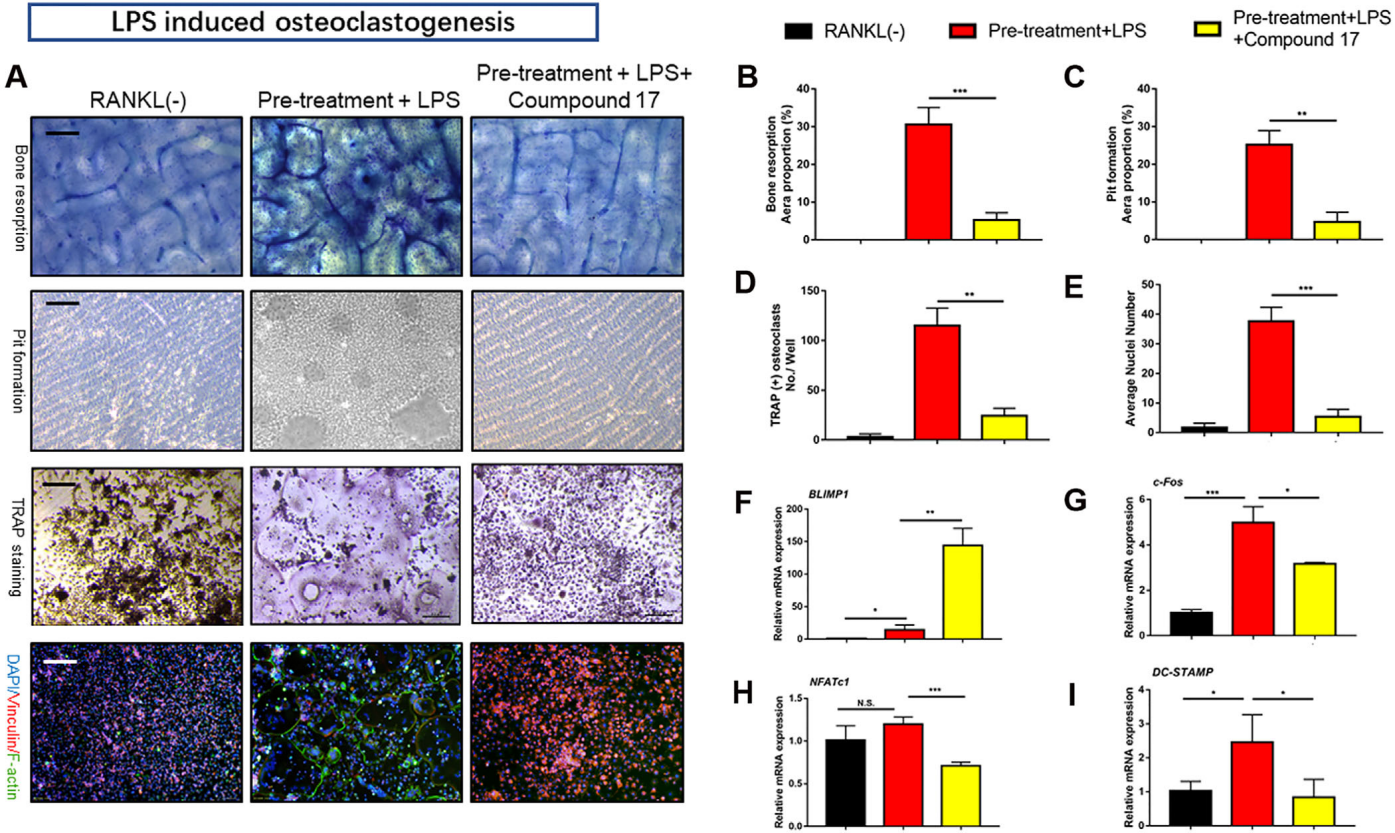
**Compound 17 suppresses LPS‐induced osteoclastogenesis and bone erosion ability**. A, Representative images of TRAP staining, FAK staining, bone resorption assay and pit formation assay of LPS induced osteoclasts in the absence or presence with compound 17 for other days. Bar represents 200 μm. Images are representative of n = 3 independent experiments. B, Quantification analysis of the proportion about bone resorption area. C, Quantification analysis of the proportion about pit formation area. D, Quantification analysis of the TRAP positive osteoclasts in each well. E, Quantification analysis of the average nuclei number in each osteoclast. F, Relative expression of marker genes in the procedure of LPS induced osteoclastogenesis from monocytes to mature osteoclasts at mRNA level. The data in the figures represent the averages ± SD. Significant differences are indicated as * (*P* < .05), ** (*P* < .01) and *** (*P* < .001) paired using Student's *t*‐test unless otherwise specified. N.S. represented as the no significant difference

LPS has been shown to play an essential role in the poor progression of inflammatory osteolysis in inflammatory models in vitro.^28^ Thus, we established an LPS‐induced OC differentiation cellular model to determine the effects of compound 17 on inflammatory osteoclastogenesis. After stimulation with RANKL and M‐CSF for 1 day, pre‐OCs were obtained and then treated with LPS. We then performed TRAP and FAK staining, which showed dramatic inhibition of OC fusion and differentiation after treatment with compound 17 during the LPS induced osteoclastogenesis. The number of TRAP‐positive OCs and F‐actin formation were also suppressed after treatment with compound 17 (Figures 3A, 3D and 3E). Bone resorption activity was also measured by using the Osteo Assay Surface and bovine cortical bone slices, and the resorbed areas per OC were significantly repressed by compound 17, which was consistent with the TRAP and FAK staining results (Figure 3A‐C). Interestingly, once LPS‐stimulated osteoclastogenesis was activated, several genes were conspicuously modulated, including NFATc1, DC‐STAMP, BLIMP1 and c‐Fos. Thus, the effects of compound 17 on the LPS‐induced expression of these genes were detected by quantitative RT‐PCR. The results revealed that the expression of these OC marker genes was increased by RANKL stimulation but significantly attenuated by compound 17 treatment (Figure 3F‐I).

### 3.5 Compound 17 impaired NF‐κB p65 nuclear translocation as well as related signaling pathway

**FIGURE 4:**
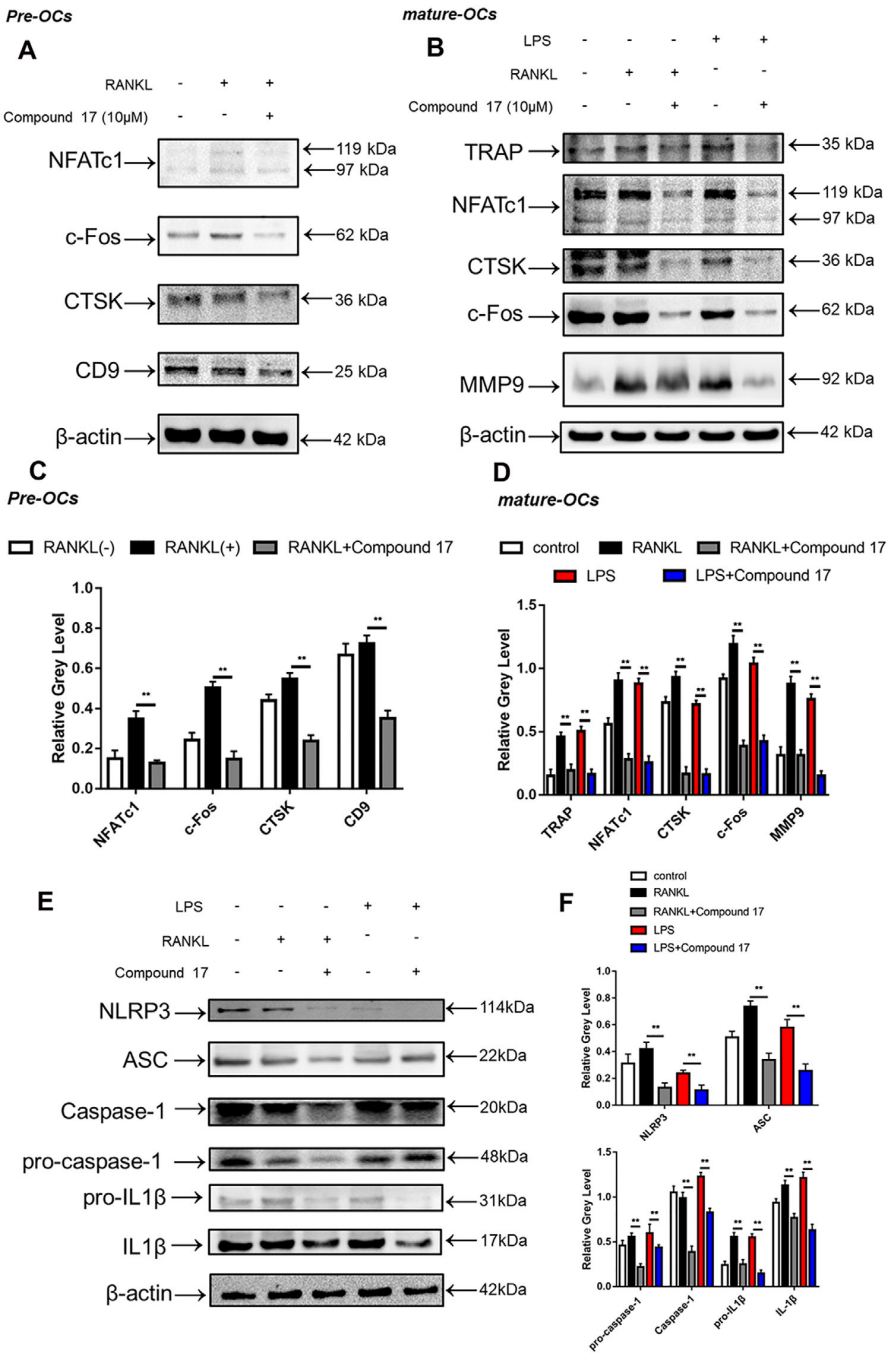
Compound 17 restrains the expression of marker genes at different stages during osteoclastogenesis and NLRP3 inflammasome signaling pathway. A, Relative expression of CTSK, CD9, c‐Fos and NFATc1 during treatment with RANKL in the presence or absence of compound 17 during the differentiation procedure from monocytes to pre‐osteoclasts at the protein level. β‐actin was used as an internal control. B, Relative expression of TRAP, CTSK, MMP9, c‐Fos and NFATc1 during treatment with RANKL in the presence or absence of compound 17 during the differentiation procedure from monocytes to mature osteoclasts at the protein level. β‐actin was used as an internal control. C, Quantification of the ratios of band intensity of CTSK, CD9, c‐Fos and NFATc1 relative to β‐actin. D, Quantification of the ratios of band intensity of TRAP, CTSK, MMP9, c‐Fos and NFATc1 relative to β‐actin. E, Relative expression of ASC, caspase‐1, pro‐caspase‐1, IL‐1β, pro‐IL1β and NLRP3 after treatment with RANKL in the presence or absence of compound 17 during the differentiation procedure from monocytes to mature osteoclasts at the protein level. β‐actin was used as an internal control. F, Quantification of the ratios of band intensity of ASC, caspase‐1, pro‐caspase‐1, IL‐1β, pro‐IL1β and NLRP3 relative to β‐actin. The data in the figures represent the averages ± SD. Significant differences are indicated as ** (*P* < .01) paired using Student's *t*‐test unless otherwise specified

**FIGURE 5:**
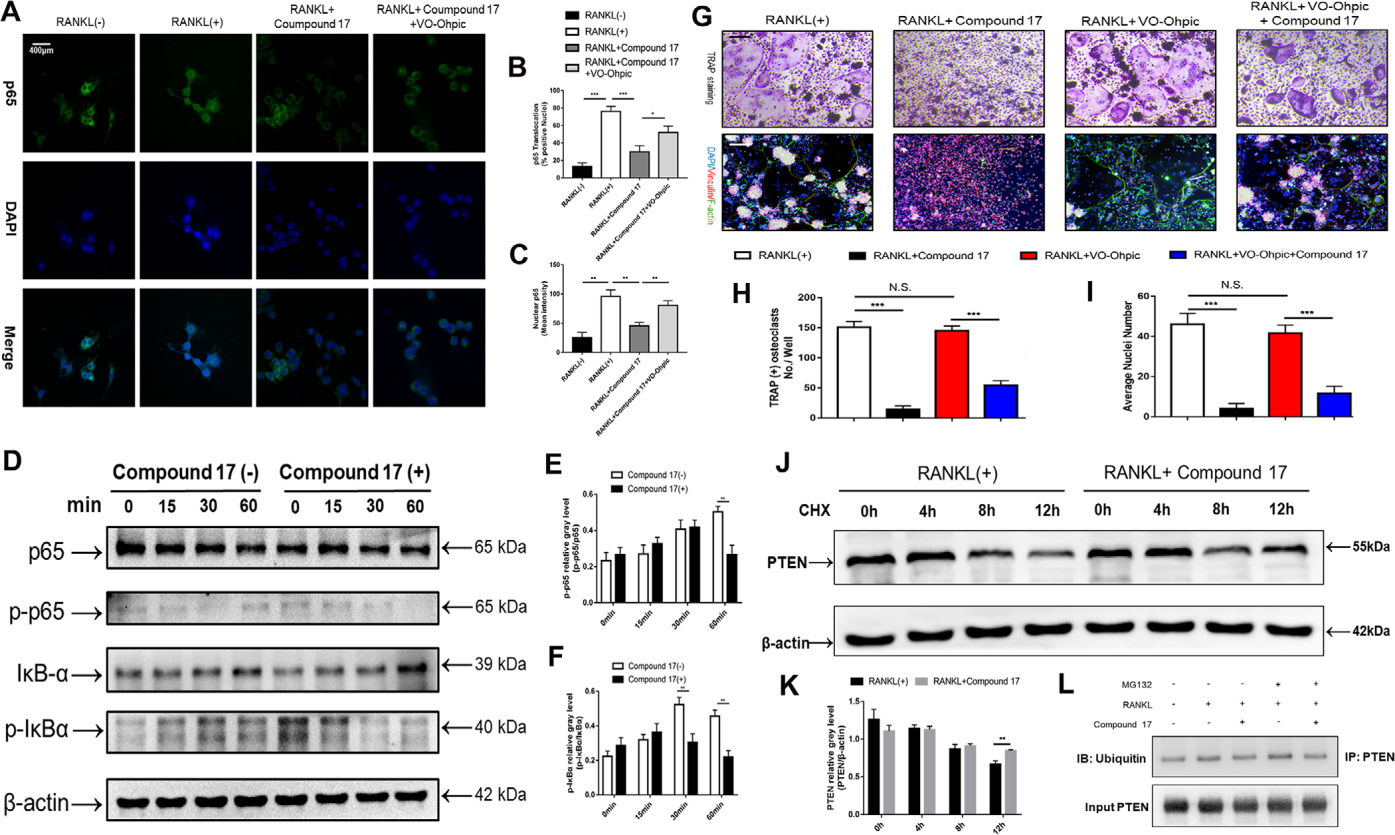
Compound 17 dramatically attenuates NF‐κB signaling pathway via the ubiquitination of PTEN. A, Representative images of the intracellular location of the NF‐κB p65, which were observed by immunofluorescence staining using confocal microscopy. Scale bar = 400μm. Images are representative of n = 3 independent experiments. B, Quantification analysis about the proportion of NF‐κB p65 nuclear translocation positive cells per well. C, Quantitative analysis of the mean intensity of NF‐ĸB p65 in the cells nuclear. D, Relative expression of p‐NFκB p65, NF‐κB p65, p‐IκBα and IκBα after treatment with RANKL in the presence or absence of compound 17 during the stimulation for 0‐60 minutes at the protein level. β‐actin was used as an internal control. E, Quantification of the ratios of band intensity of p‐NFκB p65 relative to NF‐κB p65. F, Quantification of the ratios of band intensity of p‐IκBα relative to IκBα. G, Representative images of TRAP staining and FAK staining of RANKL‐induced osteoclasts in different groups (RANKL, RANKL+Compound 17, RANKL+VO‐Ohpic, RANKL+Compound 17+VO‐Ohpic) for other days. Bar represents 200 μm. Images are representative of n = 3 independent experiments. H, Quantification analysis of the TRAP positive osteoclasts in each well. I, Quantification analysis of the average nuclei number in each osteoclast. J, BMMs were cultured with RANKL (50 ng/mL) in the presence or absence of compound 17 for 8 hours, and treated with CHX (50 μg/mL) for the indicated times. Total cell lysates were subjected to immunoblotting analysis. K, Quantification of the ratios of band intensity of PTEN relative to β‐actin. L, BMMs were pretreated with compound 17 for 8 hours, with or without MG132 (50 μM) for 2 hours, and then treated with RANKL (50 ng/mL) for 30 minutes. The cell lysates were immunoprecipitated with PTEN antibody and immunoblotted with ubiquitin and PTEN antibodies. The data in the figures represent the averages ± SD. Significant differences are indicated as ** (*P* < .01) and *** (*P* < .001) paired using Student's *t*‐test unless otherwise specified. N.S. represented as the no significant difference

To elucidate the potential mechanisms underlying compound 17‐mediated inhibition of osteoclastogenesis and bone resorption, we further investigated the main signalling pathways involved in the RANKL/RANK signalling cascade. Immunoblotting analysis showed that RANKL stimulation elevated the protein level of NFATc1 during the different stages of osteoclastogenesis, whereas compound 17 strongly inhibited the protein expression of NFATc1 (Figure 4A‐D). The inhibitory effects of compound 17 were also observed during LPS‐induced osteoclastogenesis. Additionally, we observed that compound 17 impaired RANKL‐ and LPS‐induced protein expression of c‐Fos, CTSK, MMP9 and TRAP (Figures 4B and 4D). Many reports have shown that the NF‐κB signalling pathway participates in OC formation and function.^29^ To explore the effects of compound 17 on the NF‐κB pathway in the presence of RANKL stimulation, we measured the protein levels of IκB‐α (an inhibitor of NF‐κB), p‐IκBα, p65 and p‐p65. Compound 17 restrains the RANKL‐induced phosphorylation of p65 at 60 minutes (Figure 5D‐F). As expected, RANKL‐induced IκBα phosphorylation and degradation were also significantly suppressed by compound 17 (Figure 5D‐F). Similarly, the colocalization of the NF‐κB p65 protein and nucleus were consistent with the previous reports.^28^ Immunofluorescence analysis showed that compound 17 markedly suppressed the nuclear translocation of p65, which was consistent with the inhibition of expression of these NF‐κB signalling pathway proteins (Figure 5A‐C). Meanwhile, the expression of NF‐κB p65 in the nucleus could be dramatically negatively regulated after treating with compound 17 (Figure S7).

### 3.6 Compound 17 suppressed the formation of NLRP3 inflammasome and the release of IL‐1β

Increased formation of the NLRP3 inflammasome has been shown to play an essential role in osteoclastogenesis and inflammatory osteolysis.^30^ The NLRP3 inflammasome can also be activated independently by PAMPs and DAMPs.^31^ IL‐1β in turn can be released after the formation of the NLRP3 inflammasome, which can also stimulate OC differentiation and bone resorption directly and indirectly through the induction of osteoclastogenic factors (e.g., RANKL).^32^ Bone degradation products released during this accelerated bone turnover activate the inflammasome. We found anti‐inflammatory effects of compound 17 through inhibition of the NF‐κB signalling pathway. Then, we further investigated whether the formation of the NLRP3 inflammasome and the release of IL‐1β were also negatively regulated by compound 17. As expected, the enhancement of NLRP3 and IL‐1β protein expression after RANKL and LPS stimulation was significantly attenuated after treatment with compound 17 (10 μM) (Figure 4E,F). These suppressive effects were consistent with the inhibition of the NF‐κB signalling pathway. Our findings revealed that compound 17 inhibited the activation of NLRP3 inflammasome and IL‐1β release in vitro.

### 3.7 Compound 17 inhibited osteoclastogenesis by regulating PTEN ubiquitination and degradation

PTEN has been identified as an important regulatory factor in RANKL‐induced osteoclastogenesis.^33^, ^34^ Previous studies have demonstrated that PTEN regulates the RANKL‐induced NF‐κB signalling pathway during osteoclastogenesis.^35^ Our findings suggested that compound 17 had a suppressive effect on RANKL‐ and LPS‐induced osteoclastogenesis by regulating the NF‐κB signalling pathway. Therefore, we investigated whether compound 17 regulated PTEN activity during RANKL‐induced osteoclastogenesis. To further confirm that compound 17 exerts an inhibitory effect on osteoclastogenesis through the regulation of PTEN, we used VO‐Ohpic, a PTEN inhibitor, to suppress the activity of PTEN in BMMs. VO‐Ohpic was shown to rescue the compound 17‐induced inhibition of OC formation at the concentration of 2uM. Additionally, when compound 17 was used in combination with VO‐Ohpic, its inhibitory effect on OC formation nearly disappeared (Figure 5G‐I). The inhibitory effects of NF‐κB p65 nuclear translocation and the expression of NF‐κB p65 in nucleus after administration of compound 17 were all reversed when treating with VO‐Ohpic, which showed that the compound 17 regulated NF‐κB signalling pathway in a PTEN dependent manner (Figure 5A‐C, Figure S7).

We next investigated the mechanism by which compound 17 modulated the activity of PTEN in BMMs. When the cells were treated with CHX at the concentration of 10 ug/mL, a protein synthesis inhibitor, the PTEN protein levels were slightly decreased at 4 hours and markedly reduced at 12 hours. However, compound 17 treatment inhibited PTEN degradation and sustained PTEN protein levels (Figure 5J‐K). PTEN is regulated by multiple posttranslational modifications, including ubiquitination.^36^ Moreover, compound 17 showed high similarity with the inhibitor of HECT domains. Thus, we hypothesized that PTEN might be modulated by ubiquitination, and we assessed the effect of compound 17 on PTEN ubiquitination. The results showed that compound 17 treatment led to a decrease in the ubiquitination of immunoprecipitated PTEN (Figure 5L). Taken together, these results suggest that compound 17 upregulates PTEN activity by inhibiting the ubiquitination and degradation of PTEN in BMMs, which might contribute to the inhibition of osteoclastogenesis.

### 3.8 Compound 17 promoted osteoblast differentiation and mineralization

**FIGURE 6:**
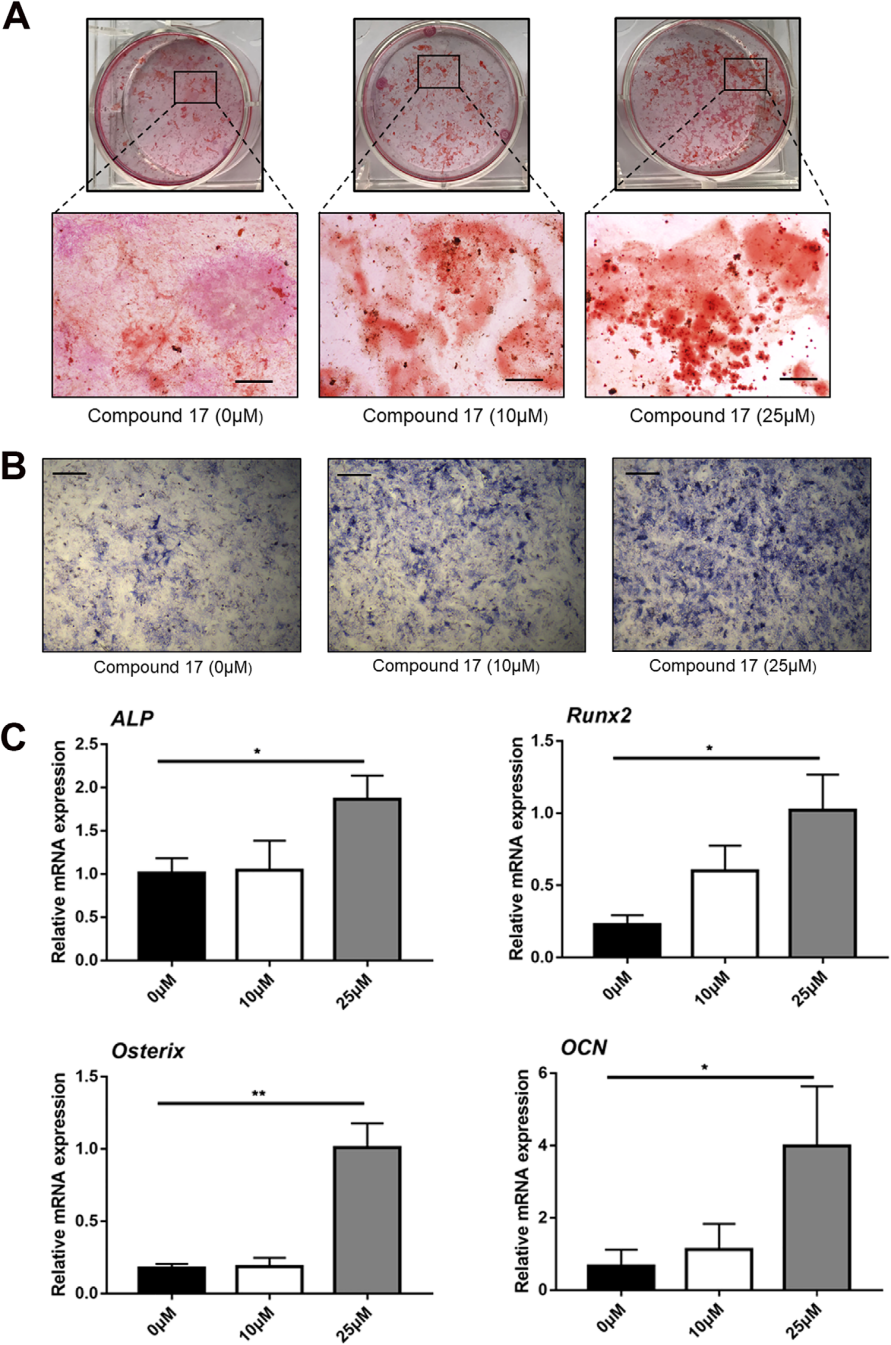
Compound 17 predominantly promotes osteoblasts differentiation and mineralization. A, Representative images of ARS staining during osteoblast mineralization in the absence or presence with compound 17 for 14 days. Bar represents 200 μm. Images are representative of n = 3 independent experiments. B, Representative images of ALP staining during osteoblastogenesis in the absence or presence with compound 17 for 7 days. Bar represents 200 μm. Images are representative of n = 3 independent experiments. C, Relative expression of marker genes during osteoblast differentiation and mineralization such as ALP, Runx2, Osterix and OCN at mRNA level. The data in the figures represent the averages ± SD. Significant differences are indicated as * (*P* < .05) and ** (*P* < .01) paired using Student's *t*‐test unless otherwise specified

OCs and osteoblasts are two key parts of bone remodelling. The dynamic balance of the bone microenvironment occurs through the cooperation of OCs and osteoblasts throughout the life of the organism. Calcium nodules are produced by osteoblasts during the mineralization of the extracellular matrix.^37^ Thus, ARS staining was performed to investigate the effects of compound 17 on osteogenic differentiation and mineralization. The results showed that the mineralization area of the plate was promoted in the compound 17‐treated groups compared to the control groups (Figure 6A). Meanwhile, the results of ALP staining also showed that compound 17 could respectively promote the ALP activity (Figure 6B). Moreover, the mRNA expression of osteoblast differentiation‐related genes, such as ALP, OCN, Osterix and Runx2, was also promoted. Taken together, these results suggest that compound 17 promotes osteoblast differentiation and mineralization (Figure 6B). Meanwhile, we also detected the effects of compounds 5 and 13 on osteogenesis by using qPCR assay. The results illustrated that they could not make obviously effects on osteogenesis (Figure S8).

### 3.9 Compound 17 dramatically modulated M1/M2 macrophage polarization and repolarization

**FIGURE 7:**
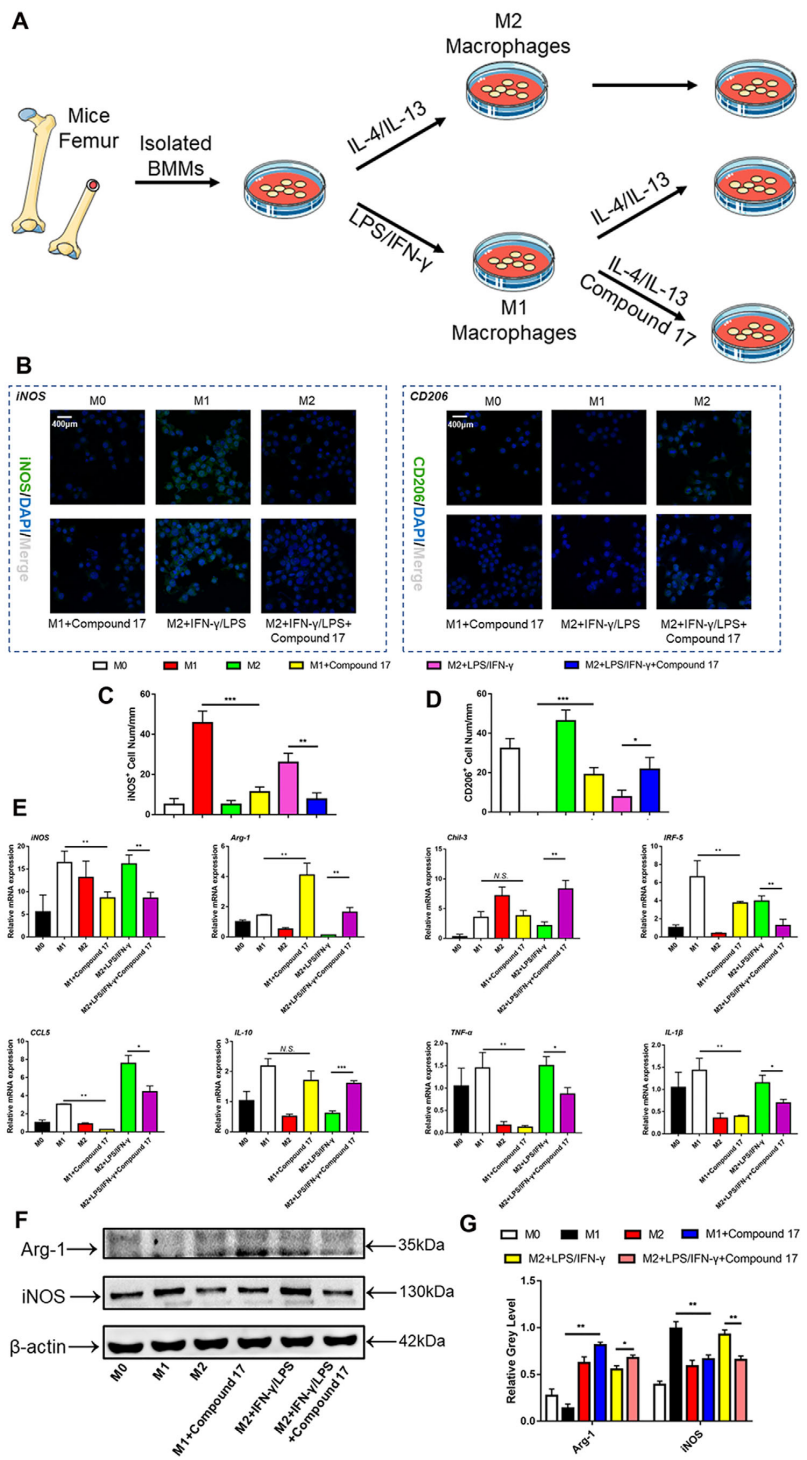
Compound 17 modulates M1 subtype macrophage polarization and repolarization. A, Schematic diagram for design of macrophage polarization and repolarization. B, Representative images of immunofluorescence staining about inducible nitric oxide synthase (iNOS) (green), CD206 (green) and DAPI (blue) in isolated BMMs among different groups. Images are representative of n = 3 independent experiments. Scale bar = 200μm. C, Quantification analysis of the iNOS positive cells per well. D, Quantification analysis of the CD206 positive cells per well. E, Relative expression of marker genes during macrophage polarization and repolarization such as iNOS, Arg‐1, Chil‐3, IRF5, CCL‐5, IL‐10, TNF‐α and IL‐1β at mRNA level. F, Relative expression of marker genes during macrophage polarization and repolarization such as (A) iNOS and Arg‐1 at protein level. G, Relative expression of iNOS and Arg‐1 after exhibiting with macrophage polarization and repolarization in the presence or absence of compound 17 at the protein level. β‐actin was used as an internal control. The data in the figures represent the averages ± SD. Significant differences are indicated as * (*P* < .05), ** (*P* < .01) and *** (*P* < .001) paired using Student's *t*‐test unless otherwise specified. N.S. represented as the no significant difference

Macrophages are a predominant part of innate myeloid cells and are involved in physiological and pathological conditions in the human body.^38^ The polarization of macrophages can be modulated by a variety of cytokines and microbial products.^39^ Macrophages can be polarized towards two distinct pathways. On the one hand, pro‐inflammatory M1 phenotype macrophages can be activated and stimulated by type 1 T helper (TH1) cytokines, such as IFN‐γ or LPS, and upregulate the expression of iNOS and induce the production of large amounts of inflammatory cytokines, such as IL‐1β and IL‐6.^40^ On the other hand, anti‐inflammatory M2 phenotype macrophages can be activated and stimulated by type 2 TH (TH2) cytokines such as IL‐4 or IL‐13, upregulate the expression of Arg‐1, Fizz‐1 and Ym‐1, and induce the production of cytokines such as IL‐10 and transforming growth factor‐β.^41^ Additionally, the polarization and repolarization of macrophages play dominant roles in inflammatory osteolysis. Excessive osteoclastogenesis could be modulated by inflammatory cytokines that are released by M1 macrophages. In our study, the M1 macrophage polarization and repolarization procedures are shown in Figure 7A. The immunofluorescence results showed that iNOS‐positive cells were negatively regulated, while CD206‐positive cells were promoted during M1 macrophage polarization and repolarization (Figure 7B‐D). In addition, the mRNA expression of TH1 and TH2 cytokines, such as iNOS, Arg‐1, Chil‐3, IRF‐5, CCL5, IL‐10, TNF‐α and IL‐1β, was regulated after treatment with compound 17 (Figure 7E). Moreover, these results also showed that Arg‐1 and iNOS were mainly modulated at the protein level (Figure 7F,G).

### 3.10 Compound 17 prevented LPS‐induced inflammatory bone destruction

**FIGURE 8:**
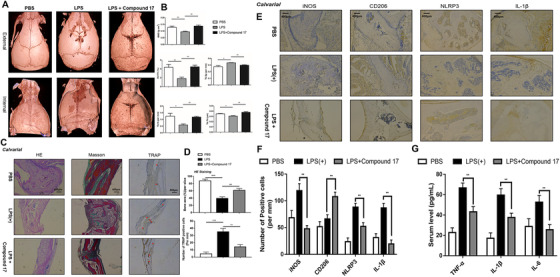
Compound 17 predominantly prevents inflammatory bone destruction in vivo. A, Representative 3D micro‐CT images of reconstructed mouse calvarial (from internal to external/from external to internal). Images are representative of n = 3 independent experiments. B, Quantification analysis of bone mineral density (BMD), trabecular number (Tb.N), trabecular separation (Tb.Sp), trabecular thickness (Tb.Th), and trabecular bone volume fraction (BV/TV). C, Representative images of cranial sections stained with H&E, Masson and TRAP from each group. Scale bars = 400μm. Images are representative of n = 3 independent experiments. D, Quantification analysis about the percentage of bone area in the border zone of the cranial H&E staining by using Image J software and the analysis of TRAP‐positive cells in each group. E, Representative images of cranial sections stained with immunohistochemical staining for iNOS, CD206, NLRP3 and IL‐1β from each group. Scale bars = 400μm. Images are representative of n = 3 independent experiments. F, Quantification analysis about the iNOS, CD206, NLRP3 and IL‐1β positive cells per mm. G, The concentration of inflammatory cytokines in the serum such as IL‐1β, IL‐6 and TNF‐α was evaluated by using ELISA assay. The data in the figures represent the averages ± SD. Significant differences are indicated as * (*P* < .05), ** (*P* < .01) and *** (*P* < .001) paired using Student's *t*‐test unless otherwise specified.

Considering the effects of compound 17 on OC and osteoblast differentiation in vitro, we next evaluated the effects of compound 17 on an inflammatory osteolysis mouse model. Moreover, compound 17 was administered at a dose of 5 mg/kg in the presence of the same dosage of LPS. Micro‐CT results confirmed that in mice, compound 17 prevented the extensive bone loss induced by LPS in the calvaria. Quantitative analysis of the calvaria further verified that compound 17 could reverse the decreases in BV/TV, BMD, Tb.Th, and Tb.N and the increase in Tb.Sp under LPS stimulation, with significant differences compared to those of the control group (Figure 8A,B). Interestingly, micro‐CT analysis of the femurs showed that the BV/TV, BMD, Tb.N and Tb.Sp were shown not the significant difference between the compound 17 group and the control group (Figure S9).

Histomorphometric analyses were performed to verify the protective effects of compound 17 on inflammatory osteolysis. Immunohistochemical about TRAP staining of the bone slices isolated from calvarial showed that compound 17 suppressed the enhancement of the OC surface/bone surface ratio and the number of OCs/bone surface area caused by LPS administration, indicating that compound 17 could restrain the activity of OCs in vivo, which was consistent with micro‐CT results (Figure 8C,D). Moreover, H&E staining showed that LPS administration dramatically reduced the percentage of bone area per slice, whereas compound 17 treatment reversed this effect (Figure 8C,D). Masson staining indicated that the area of bone tissue in each slice was also ameliorated after treatment with compound 17 (Figure 8C). However, the same inhibitory effects of compound 17 were not also observed by H&E staining and Masson staining in femur slices (Figure S10). Respectively, the IHC staining for ALP and OCN showed that treatment with compound 17 might induce osteogenesis in vivo (Figure S11).

The IHC results showed that LPS induced the production of IL‐1β and the formation of the NLRP3 inflammasome. Compound 17 almost completely reversed the LPS‐induced expression of IL‐1β and NLRP3, which was consistent with the western blot results in vitro. Interestingly, we also found that the number of iNOS‐positive cells was reduced after treatment with compound 17. Compound 17 slightly induced the expression of CD206 in combination with LPS (Figure 8E,F). Meantime, the results of ELISA about inflammatory cytokines in serum such as IL‐1β, TNF‐α and IL‐6 have shown that administration with compound 17 could restrain the release of inflammatory cytokines so as to perform anti‐inflammatory effects (Figure 8G). Overall, the in vivo results showed that compound 17 rescued LPS‐induced inflammatory osteolysis by suppressing OC activity and PTEN mediated anti‐inflammatory effects such as the suppressive activity of NLRP3 inflammasome formation and macrophage polarization.

## 4 DISCUSSION

Bone is considered a highly dynamic tissue that undergoes continuous remodelling cycles, consisting of new bone formation through osteoblast differentiation and OC‐induced bone resorption. Overactivated OCs and excessive bone resorption are involved in various kinds of musculoskeletal infection‐induced lytic bone disorders, including OM. Until now, current clinical therapies for inflammatory osteolysis have focused on anti‐inflammation and the restraint of excessive OC activity. Thus far, various studies have confirmed that therapeutic drugs, such as hormone‐like medications, bisphosphonates and denosumab, prevent excessive OC‐mediated bone resorption with several undesirable side effects.^42^, ^43^, ^44^, ^45^ Therefore, it is urgent to develop novel alternative agents to treat these diseases. In this study, we demonstrated for the first time that compound 17 (similar to the inhibitor of the HECT domain) inhibits osteoclastogenesis by repressing PTEN ubiquitination and the activities of NF‐κB and the NLRP3 inflammasome in vitro and prevents the progression of LPS‐induced inflammatory bone destruction in a mouse model in vivo.

The ubiquitin‐dependent proteolysis system plays an important role in mediating bone remodelling.^45^ A large number of E3 ubiquitin ligases contain RING domains, and approximately 28 contain HECT domains.^46^, ^47^ These enzymes catalyse the transfer of ubiquitin from E2 enzyme thioesters to a huge range of substrates and play crucial roles in many cellular functions.^46^, ^47^ Heclin has been reported to act as an important inhibitor of HECT ligase in cells by reacting with cysteine residue.^16^ Because the basic structure of compound 17 is a Michael acceptor system, compound 17 may exert a large amount of biological activity. The Michael acceptor system exerts antioxidant and anti‐inflammatory effects through covalent bonds with cysteine residues.^16^ Thus, we synthesized many compounds that are similar to the chemical structure of heclin. After comparing these compounds, compound 17 showed the highest similarity with heclin and could strongly inhibit the HECT domain via a Michael addition reaction.

First, to further elucidate the biological function of compound 17, OC differentiation and bone resorption assays were carried out. Compound 17 significantly inhibited OC differentiation in a time‐ and dose‐dependent manner. F‐actin rings were stained and visualied, indicating that compound 17 has an inhibitory effect on OC fusion. The pit formation assay demonstrated that compound 17 suppressed osteoclastic resorption both in a time‐ and dose‐dependent manner, indicating that compound 17 had an effect on OC differentiation and resorption functions.

Accumulating evidence has shown that RANKL/RANK signalling plays a dominant role in the differentiation, function and survival of OCs.^48^ The NF‐κB signalling pathway is activated after binding RANK to its ligand RANKL.^5^ The NF‐κB signalling cascade is initiated by the phosphorylation of IκB kinases (IKKs), which are activated by the RANK/TRAF6/TAK1 complex. Inactive NF‐κB dimers are retained in the cytoplasm by inhibitory IκB, and activated IKKs catalyse the phosphorylation and subsequent degradation of IκB. This process releases NF‐κB p65, which is then translocated to the nucleus to promote the transcription of NFATc1.^49^ In this study, we observed that compound 17 inhibited RANKL‐induced IκBα phosphorylation and degradation, thereby leading to the inhibition of NF‐κB p65 phosphorylation and nuclear translocation.

NFATc1 has been reported to be the dominant transcriptional regulator of OC differentiation, and its self‐amplification maintains its robust expression. Several lines of evidence show the critical role of NFATc1 in OC formation and function. Specifically, a lack of NFATc1 leads to the failure of OC formation from embryonic stem cells, and NFATc1 disruption in haematopoietic cells results in increased bone mass and decreased OCs in a mouse model.^28^ NFATc1 is the master transcription factor that regulates the expression of OC‐related genes, including DC‐STAMP, TRAP, CTSK, DC‐STAMP and CTR.^50^ In addition, our results demonstrated that compound 17 reduced the mRNA and protein expression of NFATc1, which correspondingly suppressed the expression of its downstream genes, including TRAP, CD9, Sirt‐1, OC‐STAMP, CTSK, DC‐STAMP, CTR and MMP‐9. Moreover, LPS‐induced osteoclastogenesis was also negatively regulated, which was shown by TRAP staining, FAK staining, bone resorption assays and pit formation assays. In addition, the mRNA expression of BLIMP‐1, NFATc1, DC‐STAMP and c‐Fos was also regulated in the presence of compound 17. Moreover, ARS staining showed that compound 17 induced bone matrix mineralization. Additionally, osteoblast differentiation‐related genes, such as ALP, Runx2, Osterix and OCN, were also promoted in the presence of different concentrations of compound 17.

Accumulating evidence indicates that the NF‐κB signalling pathway plays an essential role in the formation of the NLRP3 inflammasome. Activation of the NLRP3 inflammasome during osteoclastogenesis may contribute to excessive bone loss, and the inflammasome is composed of NLRP3, ASC and caspase‐1, which is a major innate immune sensor.^51^ This complex recognises diverse stimuli, such as pathogen‐associated molecular patterns and endogenous damage‐associated molecular patterns. Upon activation, the NLRP3 inflammasome leads to caspase‐1 activation, which is responsible for the maturation of IL‐1β and IL‐18. In particular, IL‐1β is an important mediator of inflammasome‐related metabolic diseases.^51^ The pathological involvement of IL‐1β in bone destruction has been reported. Moreover, IL‐1β mediates RANKL‐induced OC differentiation in vitro.^52^ Additionally, IL‐1β participates in M1 macrophage polarization and repolarization in inflammatory conditions.^53^ Intriguingly, further investigation into the mechanisms revealed that compound 17 also affected RANKL‐ and LPS‐induced osteoclastogenesis through the regulation of NLRP3 inflammasome formation and the release of IL‐1β, which illustrated that compound 17 had an inhibitory effect on OC differentiation in an NLRP3‐ and IL‐1β‐dependent manner. These findings also showed that M1 macrophage polarization and repolarization could also be negatively modulated.

PTEN is a multifunctional molecule that is expressed in various types of cells and regulates multiple cellular processes, such as cell proliferation, survival, adhesion, motility and apoptosis.^35^, ^36^ The major function of PTEN is to negatively regulate several signalling pathways, such as the NF‐κB pathway.^54^ In recent studies, PTEN was identified as an important regulatory factor in RANKL‐induced osteoclastogenesis.^35^ The overexpression of PTEN inhibits RANKL‐induced OC formation by negatively regulating RANKL‐induced NF‐κB signalling.^35^ In our study, we observed that compound 17 could promote the expression of total PTEN in BMMs during osteoclastogenesis. Therefore, the compound 17‐mediated inhibition of NF‐κB signalling pathways could be modulated due to enhanced PTEN activity. Moreover, when we used VO‐Ohpic, a PTEN inhibitor, to suppress the activity of PTEN, the compound 17‐mediated inhibition of OC formation was rescued, suggesting that the inhibitory effect of compound 17 on OC formation is PTEN‐dependent. In this study, we used compound 17 (an HECT ligase inhibitor) and observed a decrease in PTEN protein levels within 12 hours in the presence of CHX, whereas PTEN protein levels were maintained in cells treated with compound 17, indicating that compound 17 regulates PTEN protein levels by suppressing degradation rather than promoting synthesis. Furthermore, our results showed that compound 17 inhibits PTEN ubiquitination, which could be the cause of the reduced degradation of PTEN and elevated PTEN protein levels.

**FIGURE 9:**
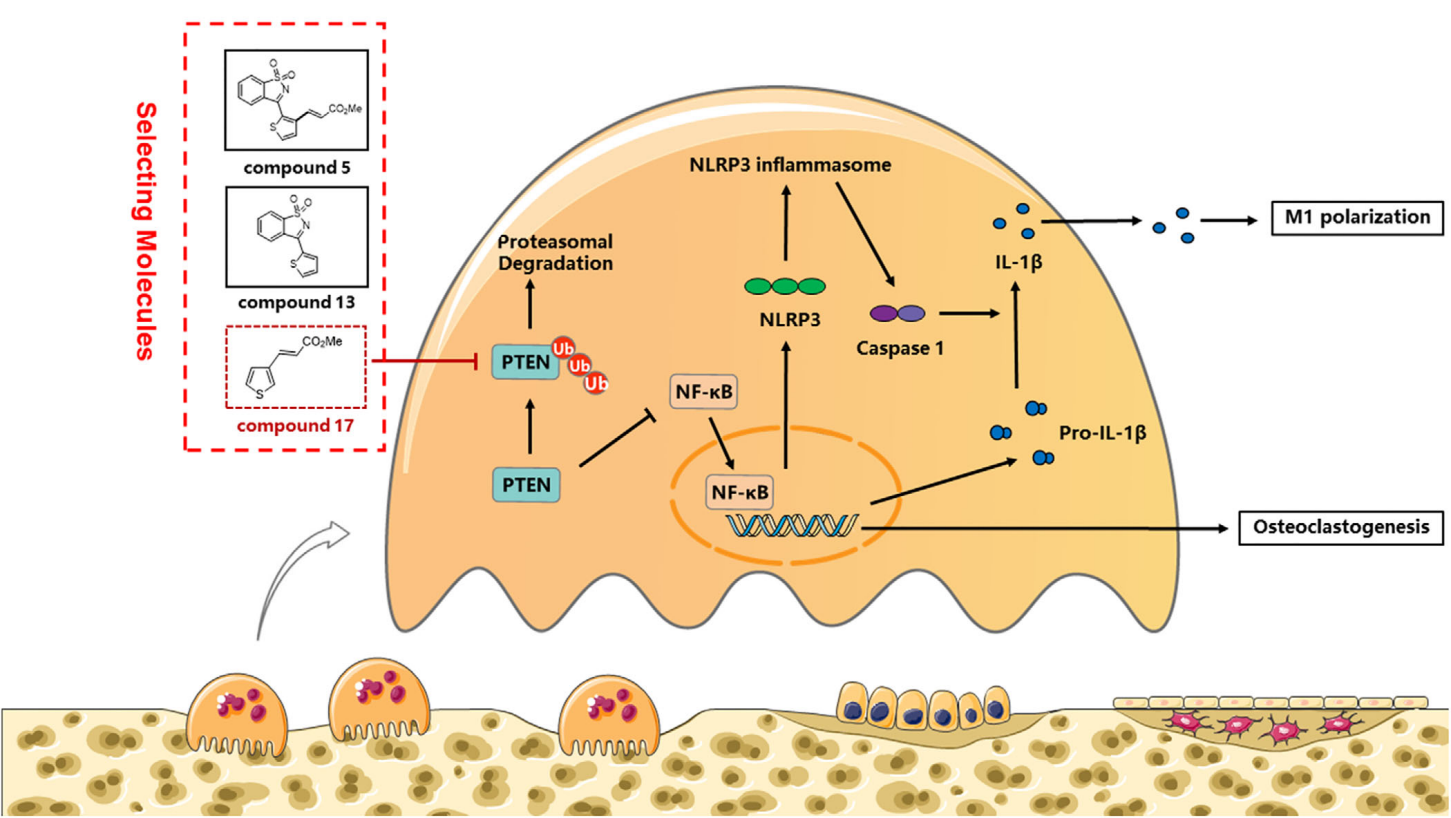
**A promising scheme for the protective effects of compound 17 on inflammatory bone destruction**. Schematic diagrams showing that compound 17 makes the protective effects on inflammatory osteolysis through negatively regulating excessive osteoclastogenesis via PTEN dependent signaling

In summary, our study demonstrated for the first time that compound 17 (similar to the inhibitor of the HECT domain) suppressed osteoclastogenesis in vitro by inducing PTEN activity by restraining PTEN ubiquitination, thus inhibiting the RANKL‐induced NF‐κB pathway. Moreover, RANKL could also promote bone mineralization during osteoblastogenesis in the presence of various doses of compound 17. Furthermore, the polarization and repolarization of M1 macrophages were also negatively modulated. In vivo experiments showed that compound 17 prevented LPS‐induced inflammatory osteolysis by inhibiting OC activity, inhibiting M1 macrophage polarization and repolarization and the formation of the NLRP3 inflammasome (Figure 9). The limitation about our study showed that the high concentration of compound 17 has been used in vitro experiments, which might contribute to the problem of clinical translation. Meanwhile, the Michael addition reactions could also be occurred in the presence of compound 17. Above all these findings, we have confirmed that compound 17 might act as a potential novel drug to treat inflammatory osteolysis‐related diseases in the future after improving the related Michael addition reactions.

## ETHICS APPROVAL AND CONSENT TO PARTICIPATE

The study protocol was approved by ethical committee of Third military medical University.

## AVAILABILITY OF DATA AND MATERIALS

The data sets used in the current study are available from the corresponding author on reasonable request.

## CONFLICT OF INTEREST

The authors declare that there is no conflict of interest that could be perceived as prejudicing the impartiality of the research reported.

## AUTHOR CONTRIBUTIONS

Study concept and design: Chen, Wang and Dong. Acquired data: Chen, Yang, Hu, Li, Tang, Li, Zhang and Dong. Analysis and interpretation of data: Chen, Li, Hu, Wang, Wei and Dong. Drafting and critical revision of the manuscript for important intellectual content: Chen, Wang, Wei and Dong. Drew figures: Chen, Hu and Dong. Supplied the significant technical and material for our experiments: Li, Yang, Wei and Dong. All authors read and approved the final manuscript.

## Supporting information

Table S1 Primer sequences for qPCR
Figure S1 The chemical synthetic route and procedure of compound 5
Figure S2 The nuclear magnetic hydrogen spectrum of compound 5
Figure S3 The nuclear magnetic carbon spectrum of compound 5
Figure S4 The chemical synthetic procedure and characterisation of compound 13 and compound 17 were displayed on the previous studies
Figure S5 The chemical formulas of Compound 1 to 17
Figure S6 Compound 17 could promote apoptosis rate during RANKL‐induced osteoclastogenesis
Figure S7 Compound 17 could modulate macrophage polarization in vivo at the section of femur
Figure S8 Compound 17 could negatively regulate the inflammatory osteolysis in vivo at the section of femur
Figure S9 The micro‐CT analysis of femur during LPS‐induced calvarial inflammatory osteolysis.
A, Representative 3D micro‐CT images of reconstructed mouse femur. Images are representative of n = 3 independent experiments. B, Quantification analysis of bone mineral density (BMD), trabecular number (Tb.N), trabecular separation (Tb.Sp), trabecular thickness (Tb.Th), and trabecular bone volume fraction (BV/TV)
Figure S10 The expression of osteogenic genes after treating with Compound 5 and 13 during osteogenesis at mRNA level.
Figure S11 Compound 17 could dominantly up‐regulate the expression of ALP and OCN during inflammatory osteolysis in vivo at the section of femur
