# Supplementary material for: A selected small molecule prevents inflammatory osteolysis through restraining osteoclastogenesis by modulating PTEN activity
Source: Clin Transl Med. 2020 Dec 1;10(8):e240. doi: 10.1002/ctm2.240 (PMC7708775; doi:10.1002/ctm2.240)
Supplement: Supplementary file 1 — Table S1 Primer sequences for qPCR Figure S1 The chemical synthetic route and procedure of compound 5 Figure S2 The nuclear magnetic hydrogen spectrum of compound 5 Figure S3 The nuclear magnetic carbon spectrum of compound 5 Figure S4 The chemical synthetic procedure and characterisation of compound 13 and compound 17 were displayed on the previous studies Figure S5 The chemical formulas of Compound 1 to 17 Figure S6 Compound 17 could promote apoptosis rate during RANKL‐induced osteoclastogenesis Figure S7 Compound 17 could modulate macrophage polarization in vivo at the section of femur Figure S8 Compound 17 could negatively regulate the inflammatory osteolysis in vivo at the section of femur Figure S9 The micro‐CT analysis of femur during LPS‐induced calvarial inflammatory osteolysis. A, Representative 3D micro‐CT images of reconstructed mouse femur. Images are representative of n = 3 independent experiments. B, Quantification analysis of bone mineral density (BMD), trabecular number (Tb.N), trabecular separation (Tb.Sp), trabecular thickness (Tb.Th), and trabecular bone volume fraction (BV/TV) Figure S10 The expression of osteogenic genes after treating with Compound 5 and 13 during osteogenesis at mRNA level. Figure S11 Compound 17 could dominantly up‐regulate the expression of ALP and OCN during inflammatory osteolysis in vivo at the section of femur [file CTM2-10-e240-s001.pdf]

Supplementary Figures and Tables:

Supplementary Table S1. Primer sequences for qPCR

Genes	Forward	Reverse
DC-STAMP	5'-TTATGTGTTTCCACGAAGCCCTA-3'	5'-ACAGAAGAGAGCAGGGCAACG-3'
CTR	5`-CGCATCCGCTTGAATGTG-3`	5`-TCTGTCTTTCCCCAGGAAATGA-3`
MMP9	5'-ACCCGAAGCGGACATT-3'	5'-GGCATCTCCCTGAACG-3'
SIRT-1	5'-GAAGTATGACAA AGATGA-3'	5'-AGAGCTTCTTGGAGACTG-3'
CD9	5'-CGGTCAAAGGAGGTAG-3'	5'-GGAGCCATAGTCCAATA-3'
SRC	5'-AACTCCTGTCCAGCCAACCTTC-3'	5'-TCTGCCTCTCTTTAGCCAATGC-3'
OSCAR	5'-GGTCCTCATCTGCTTG-3'	5'-TATCTGGTGGAGTCTGG-3'
BLIMP-1	5'-TGCTTATCCCAGCACCCC-3'	5'-CTTCAGGTTGGAGAGCTGACC-3'
OC-STAMP	5'-GGGCTACTGGCATTGCTCTTAGT-3'	5'-CCAGAACCTTATATGAGGCGTCA-3'
CTSK	5`-GAAGAAGACTCACCAGAAGCAG-3`	5`-TCCAGGTTATGGGCAGAGATT-3`
c-Fos	5`-CGGGTTTCAACGCCGACTA-3`	5`-TTGGCACTAGAGACGGACAGA-3`
NFATc1	5`-CCCGTCACATTCTGGTCCAT-3`	5`-CAAGTAACCGTGTAGCTGCACAA-3`
iNOS	5'-GTTCTCAGCCCAACAATACAAGA-3'	5'-GTGGACGGGTCGATGTCAC-3'
Arg-1	5'-CTCCAAGCCAAAGTCCTTAGAG-3'	5'-CTCCAAGCCAAAGTCCTTAGAG-3'
Chil-3	5'-ATTCTGTGACCATCCCCTCAT-3'	5'-TGTATGTGCCTCTGAACCCAC-3'
IRF5	5'-GGTCAACGGGGAAAAGAAACT-3'	5'-CATCCACCCCTTCAGTGTACT-3'
CCL5	5'-CTACTCGGGAGGCTAAGGCAGGAA-3'	5'-GAGGGGTTGAGACGGCGGAAGC-3'
IL-10	5'-ATCCAAGACAACACTACTAA-3'	5'-TAAATATCCTCAAAGTTCC-3'
TNF-α	5'- GTCCTGCTCTACGTGACGAG-3'	5'- TCTCTCCTTTTCTGCCATCTCT-3'
IL-1β	5`-CTCAACTGTGAAATGCCACC-3`	5`-TGTCCTCATCCTGGAAGGT-3`
β-actin	5`-TCCCTGTATGCCTCTG-3`	5`- ATGTCACGCACGATTT-3`

Supplementary Figure S1:

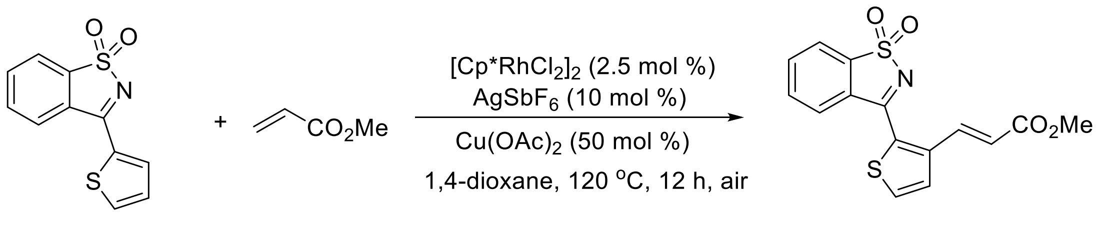

1 **2** (3 equiv) **5**, 85% yield

The chemical synthetic route and procedure of compound 5. A 10 mL of Schlenk tube equipped with a stirrer bar was charged with **1** (0.2 mmol), $[Cp^*RhCl_2]_2$ (2.5 mol%), $AgSbF_6$ (10 mol%), and Cu $(OAc)_2$ (50 mol%) under air, followed by addition of 1,4-dioxane (1 mL) and methyl acrylate **2** (0.6 mmol). Then, the Schlenk tube was sealed with a Teflon screwcap and the reaction mixture was stirred at 120 °C for 12 h. Upon cooling to room temperature, the reaction mixture was diluted with 10 mL of ethyl acetate, filtered through a pad of silica gel, followed by washing the pad of the silica gel with ethyl acetate (20 mL). Subsequently, the filtrate was concentrated under reduced pressure. The residue was purified by flash chromatography on silica gel to afford compound **5**.

Supplementary Figure S2:

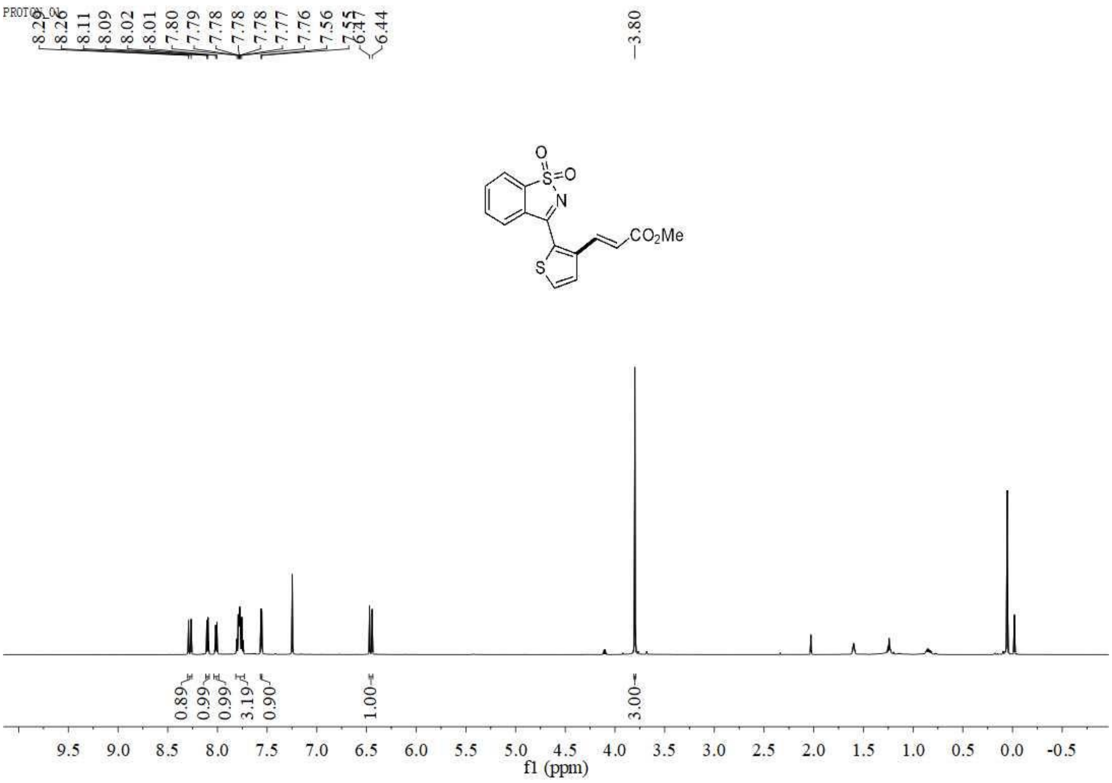

The nuclear magnetic hydrogen spectrum of compound 5. (*E*)-Methyl 3-(2-(1,1-dioxidobenzo[d]isothiazol-3-yl)cyclopenta-1,4-dien-1-yl)acrylate (5): Yellow solid (85% yield, eluent = petroleum ether /EtOAc (6:1)); Mp = 146–147 °C; **^{1}H NMR** (600 MHz, $CDCl_3$): δ 8.28 (d, J = 16.2 Hz, 1H), 8.10 (d, J = 7.2 Hz, 1H), 8.01 (d, J = 7.8 Hz, 1H), 7.82–7.73 (m,

3H), 7.56 (d, *J* = 5.4 Hz, 1H), 6.46 (d, *J* = 16.0 Hz, 1H), 3.80 (s, 3H); **^{13}C NMR** (150 MHz, $CDCl_3$): δ 166.6, 163.5, 143.4, 140.5, 136.5, 133.7, 133.6, 132.6, 130.8, 130.0, 128.1, 126.4, 123.1, 122.8, 52.0; **HRMS** (ESI) Calcd for $C_{15}H_{11}NO_4S_2$ $[M + Na]^+$ 356.0027, found 356.0030.

Supplementary Figure S3:

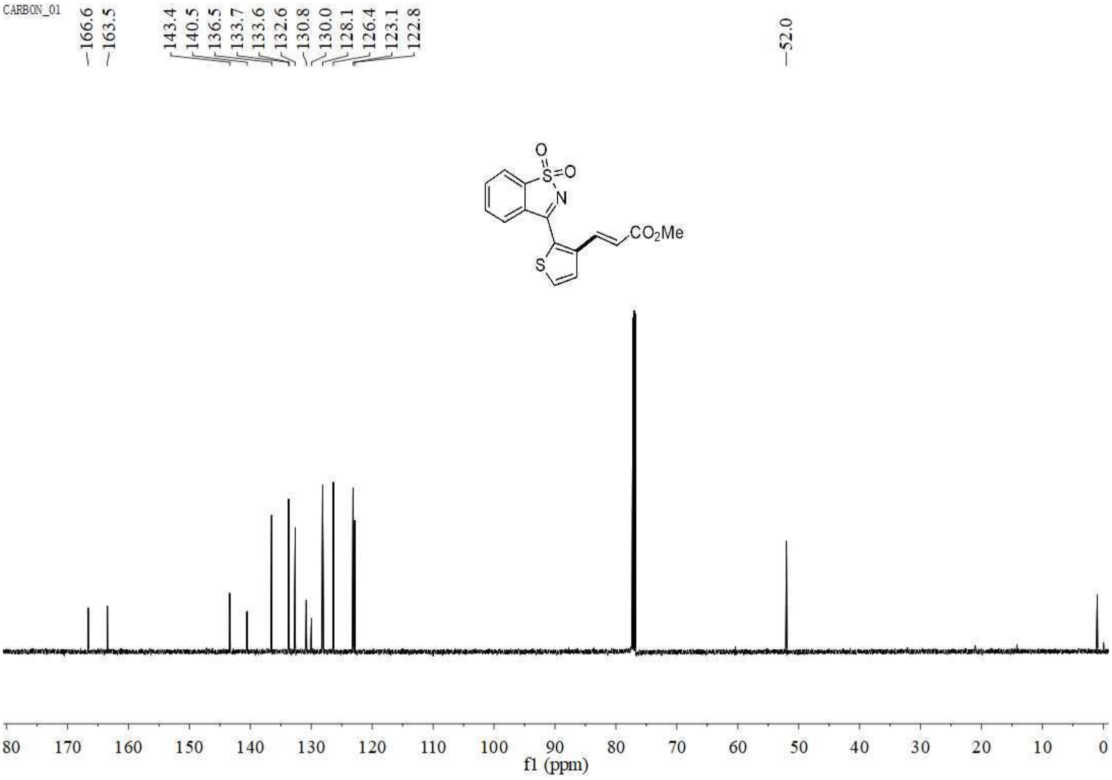

The nuclear magnetic carbon spectrum of compound 5.

Supplementary Figure S4:

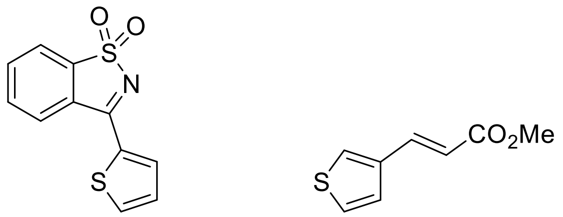

The chemical synthetic procedure and characterization of compound 13 and compound 17 were displayed on the previous studies.

(1) Nishimura, T.; Noishiki, A.; Tsui, G. C.; Hayashi, T. *J. Am. Chem. Soc.* **2012**, *134*, 5056.
(2) Xie, G.; Chellan, P.; Mao, J.; Chibale, K.; Smith, G. S. *Adv. Synth. Catal.* **2010**, *352*, 1641.

Supplementary Figure S5:

The chemical formula of these compounds 1 to 17.

**Number**	**Chemical Formula**	**Molecule weight**	**Molecule Formula**
Compound-1	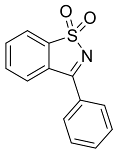	243.28	C13H9NO2S
Compound -2	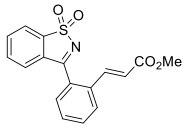	327.35	C17H13NO4S
Compound -3	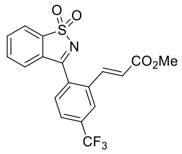	395.04	C18H12F3NO4S
Compound -4	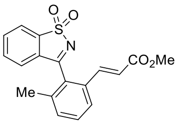	341.38	C18H15NO4S
Compound -5	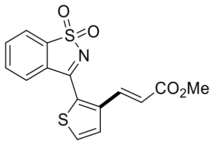	333.38	C15H11NO4S2
Compound -6	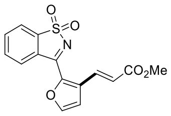	317.32	C15H11NO5S
Compound -7	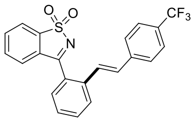	413.41	C22H14F3NO2S
Compound -8	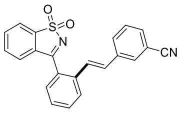	370.42	C22H14N2O2S
Compound -9	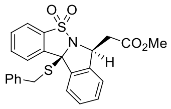	451.56	C24H21NO4S2
Compound -10	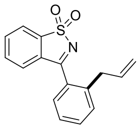	283.34	C16H13NO2S
Compound -11	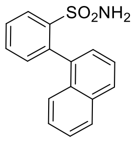	283.34	C16H13NO2S

Compound -12	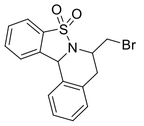	364.26	C16H14BrNO2S
Compound -13	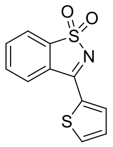	248.99	C11H7NO2S2
Compound -14	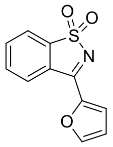	233.24	C11H7NO3S
Compound -15	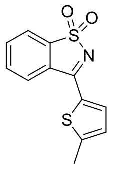	263.34	C12H9NO2S2
Compound -16	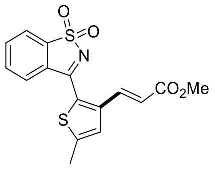	347.41	C16H13NO4S2
Compound -17	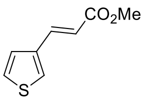	168.21	C8H8O2S

Supplementary Figure S6: **Compound 17 could promote apoptosis rate during RANKL induced osteoclastogenesis.**

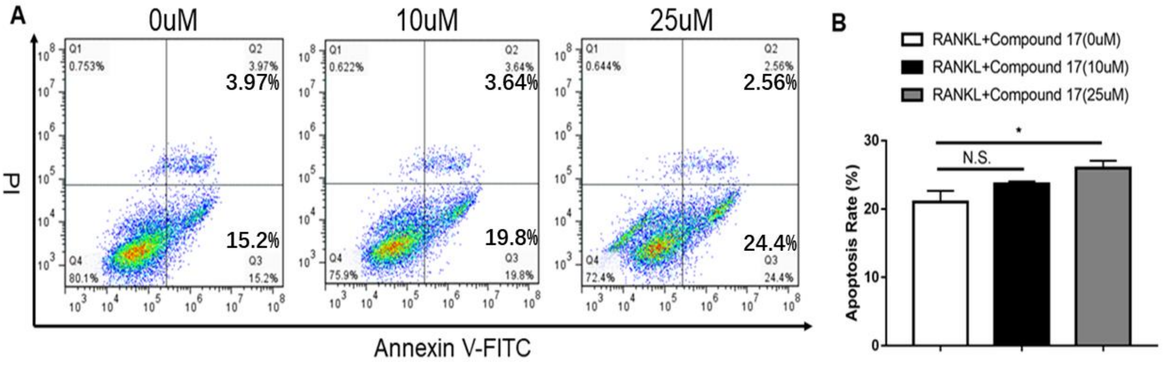

(A) Representative images of apoptosis rate during RANKL induced osteoclastegenesis in the presence of different dose of compound 17 (0μM, 10μM, 25μM).

(B) Quantification of apoptosis rate in different groups. The data in the figures represent the averages ±SD. N.S. represented as no significant difference. Significant differences are

indicated as *p<0.05 paired using Student's t test unless otherwise specified.

Supplementary Figure S7. **The expression of NF-κB p65 in the nuclear protein in the presence or absence of compound 17 during RANKL stimulation.**

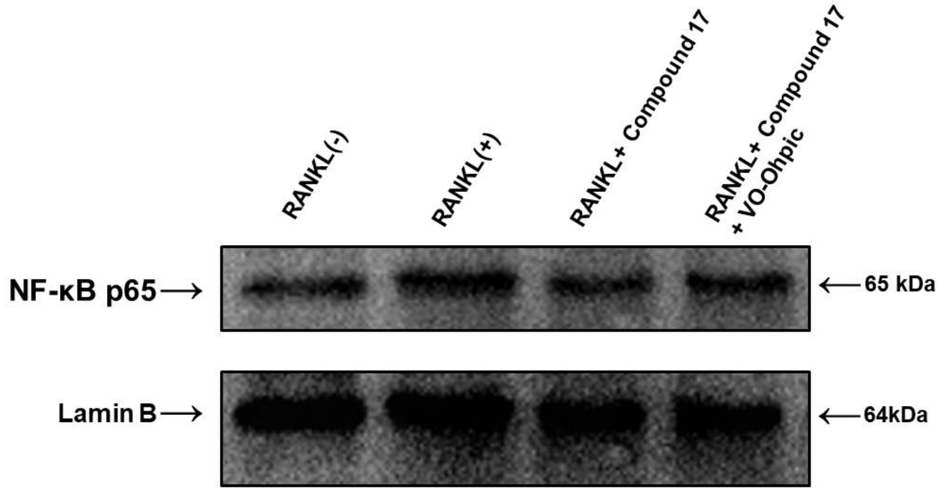

Supplementary Figure S8. **The expression of osteogenic genes after treating with Compound 5 and 13 during osteogenesis at mRNA level.**

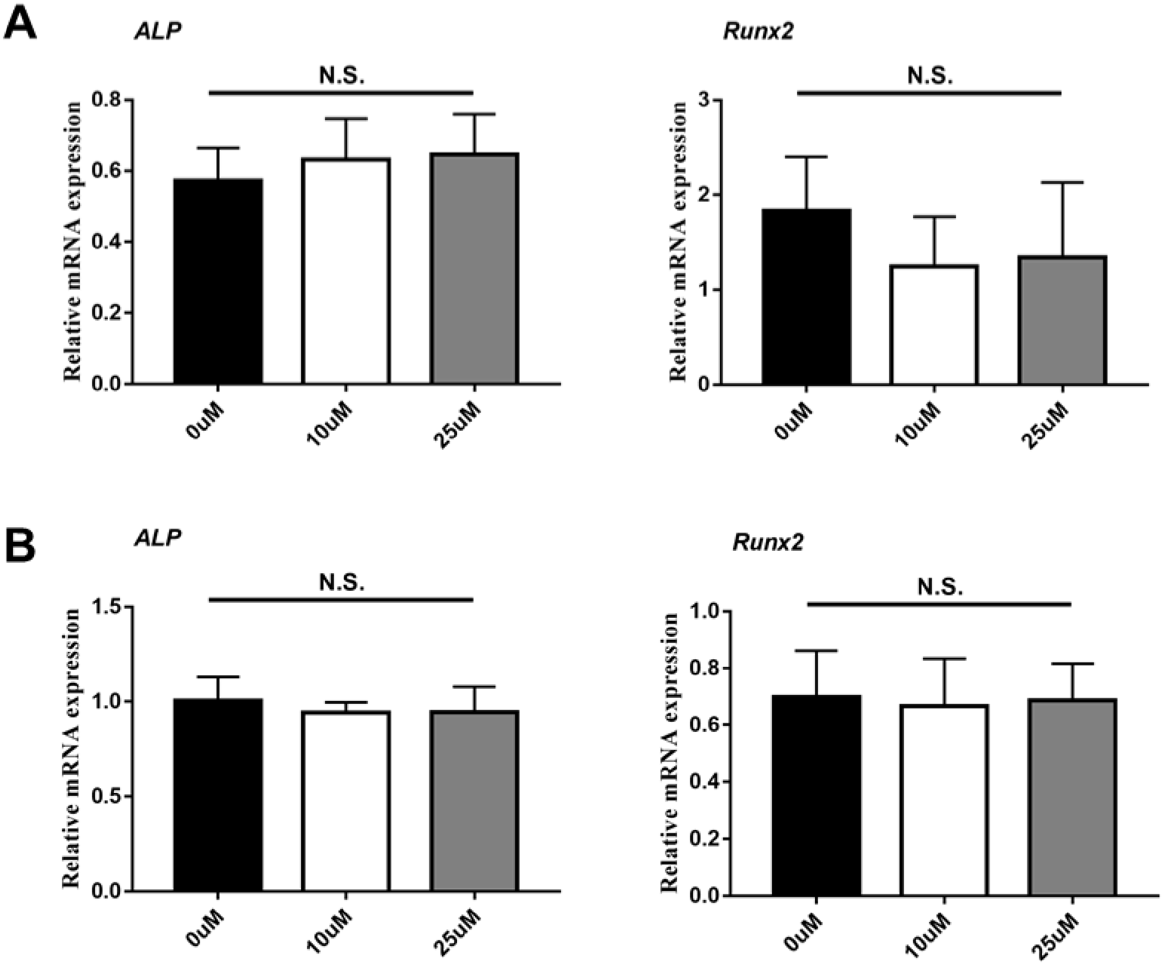

(A) Relative expression of marker genes (ALP and Runx2) during osteoblast differentiation and mineralization after administration with compound 5.

(B) Relative expression of marker genes (ALP and Runx2) during osteoblast differentiation and mineralization after administration with compound 13.

Supplementary Figure S9: **The micro-CT analysis of femur during LPS-induced calvarial inflammatory osteolysis.**

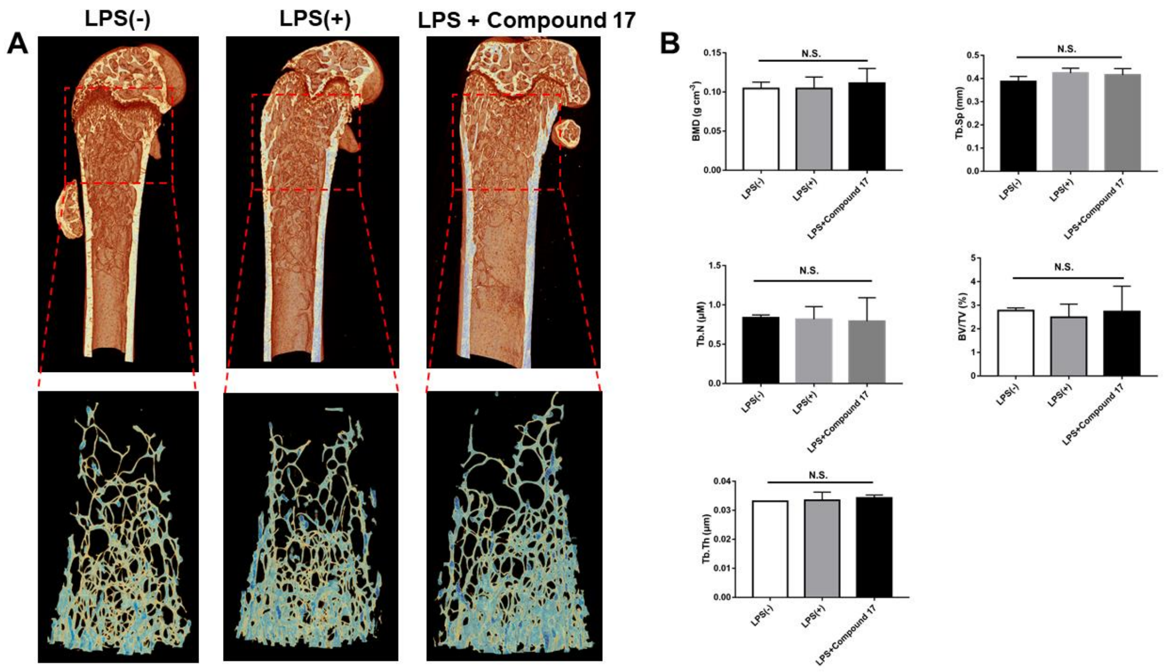

(A) Representative 3D micro-CT images of reconstructed mouse femur. Images are representative of n=3 independent experiments.

(B) Quantification analysis of bone mineral density (BMD), trabecular number (Tb. N), trabecular separation (Tb. Sp), trabecular thickness (Tb.Th), and trabecular bone volume fraction (BV/TV). Images are representative of n=3 independent experiments.

Supplementary Figure S10: **Compound 17 could negatively regulate the inflammatory osteolysis *in vivo* at the section of femur.** Representative images of HE and Masson staining at the section of femur during LPS induced inflammatory osteolysis.

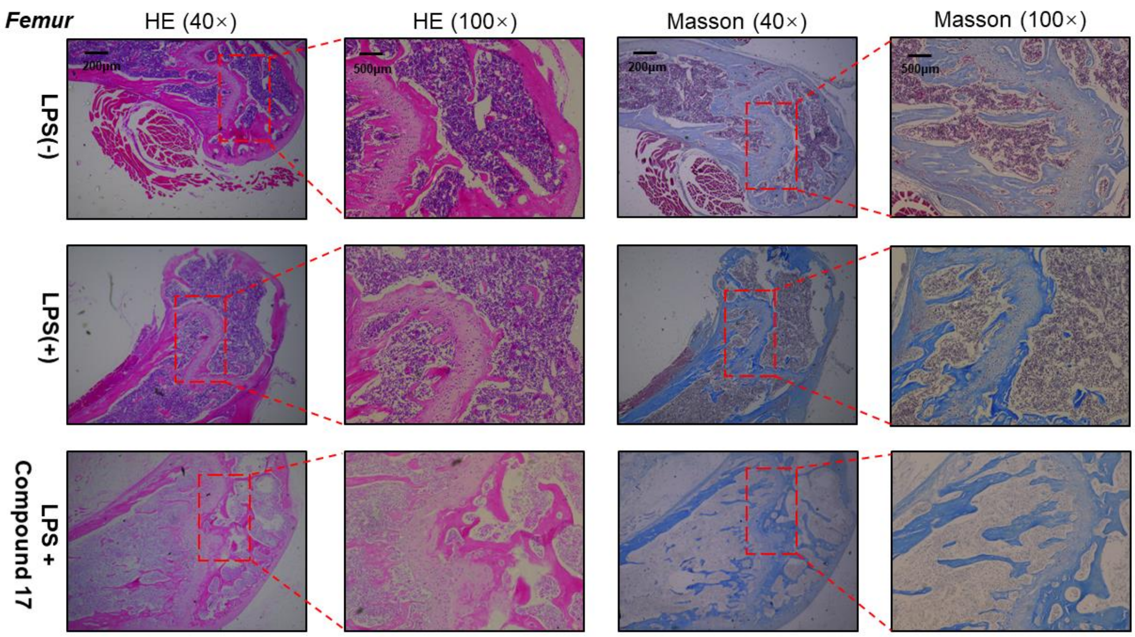

Supplementary Figure S11. **Compound 17 could dominantly up-regulate the expression of ALP and OCN during inflammatory osteolysis in vivo at the section of femur.**

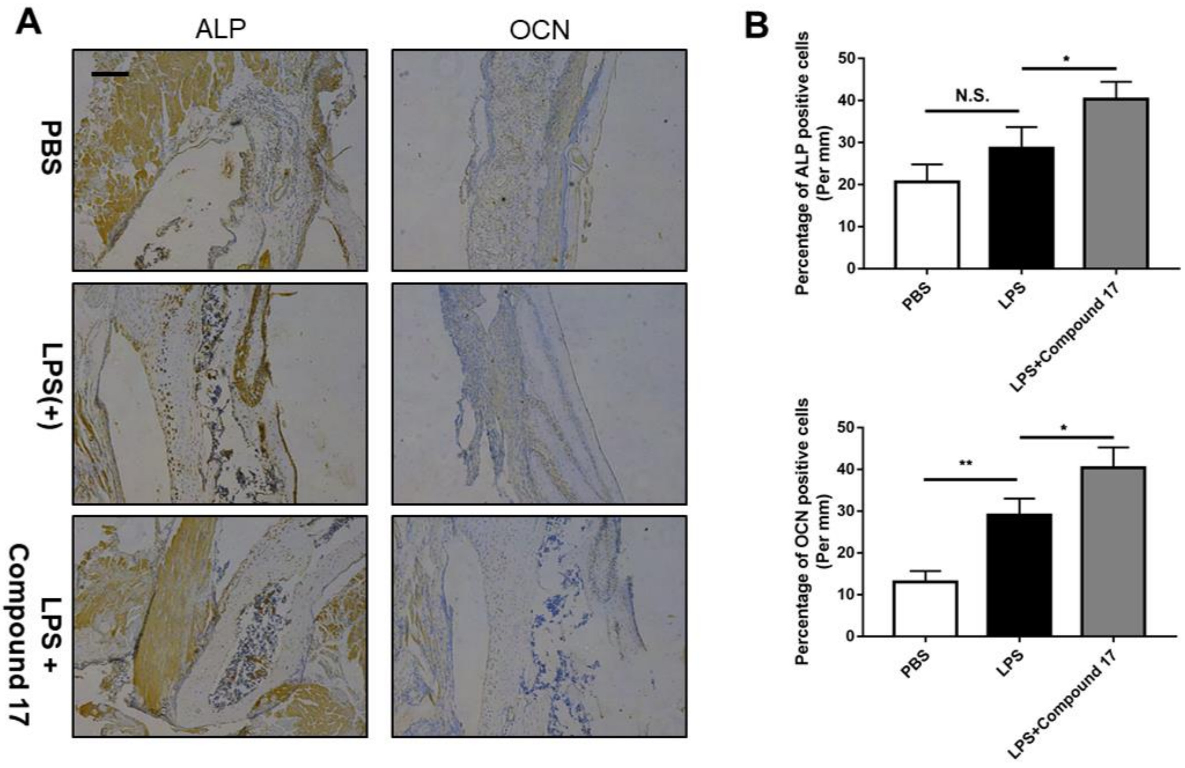

(A) Representative images of cranial sections stained with ALP and OCN from each group. Scale bars=400μm. Images are representative of n=3 independent experiments.

(B) Quantification analysis about the percentage of bone area in the border zone of the cranial ALP and OCN staining by using Image J software in each group.

Supplementary Figure S12: **Compound 17 could modulate macrophage polarization *in vivo* at the section of femur.**

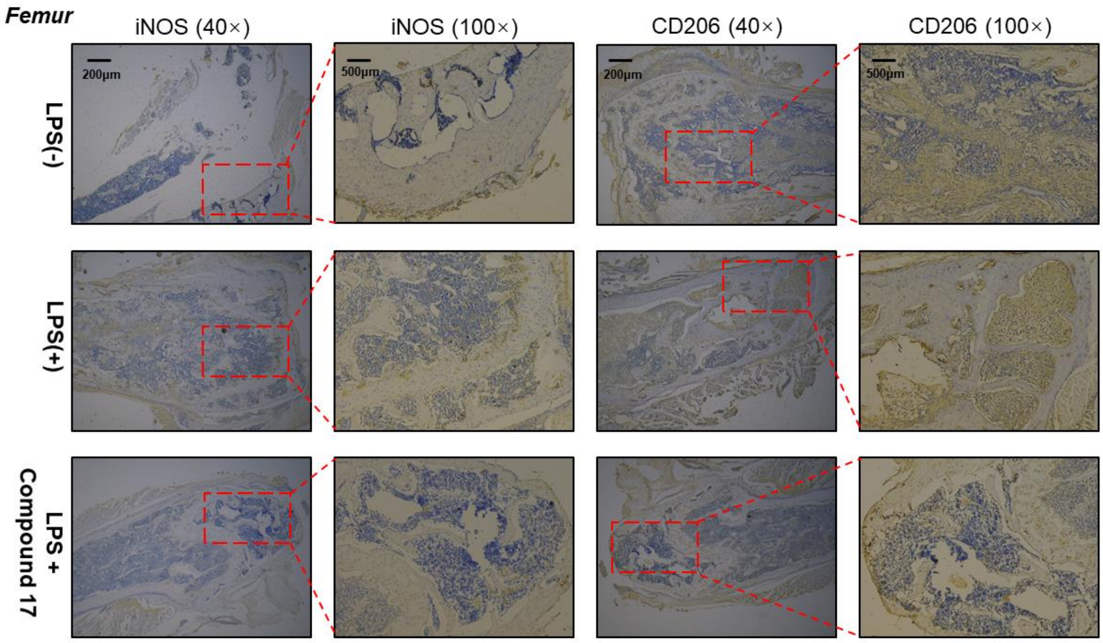

Representative images of immunochemistry staining of iNOS and CD206 at the section of femur during LPS induced inflammatory osteolysis.

Supplementary Figure S13: **The histological analysis of liver and kidney toxicity after administration with compound 17.**

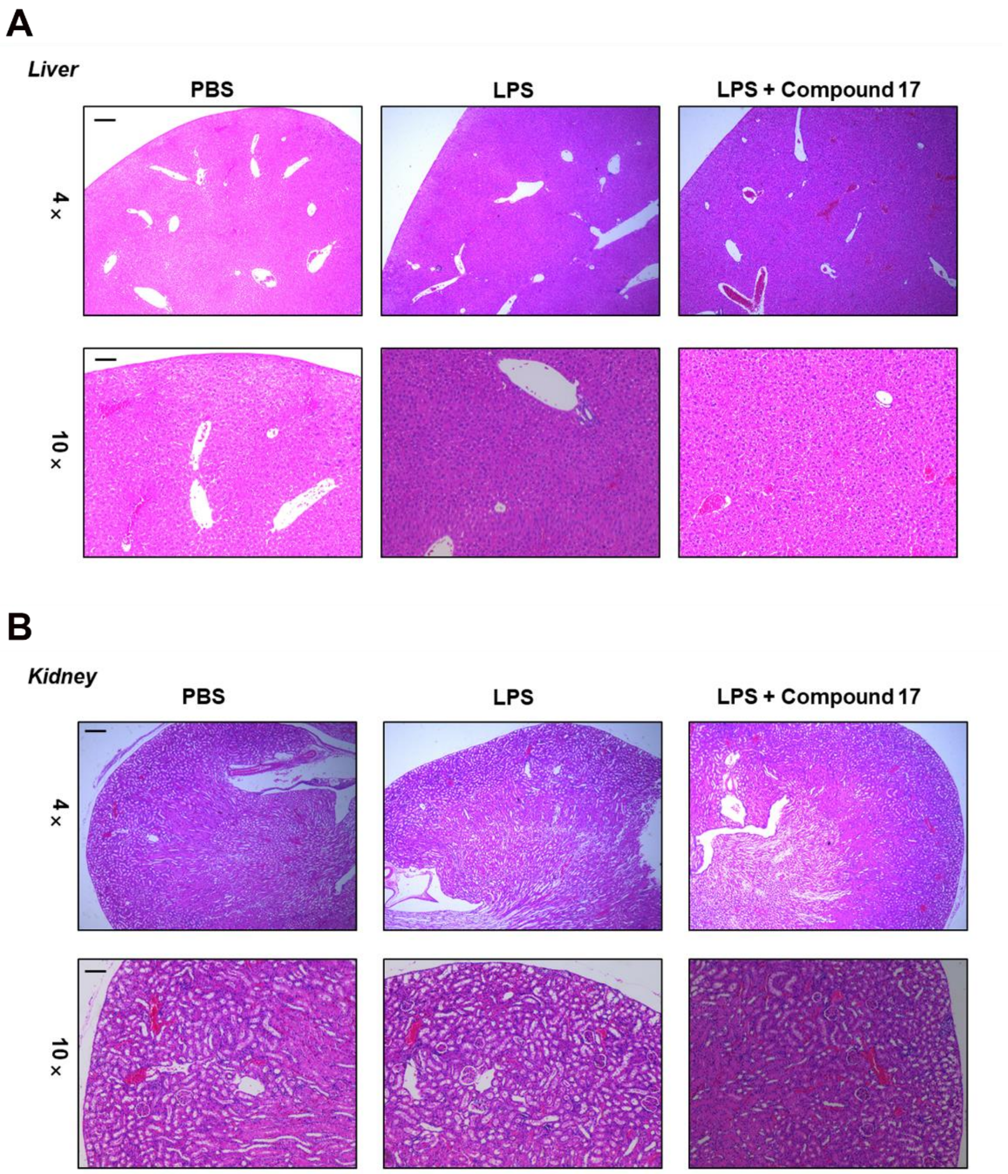

(A)Representative images of liver tissue after completing HE staining from each group. Scale bars=200μm. Images are representative of n=3 independent experiments.

(B)Representative images of kidney tissue after completing HE staining from each group. Scale bars=200μm. Images are representative of n=3 independent experiments.

Supplementary Figure S14: **Compound 17 respectively promotes the expression during osteoblasts differentiation at protein level.**

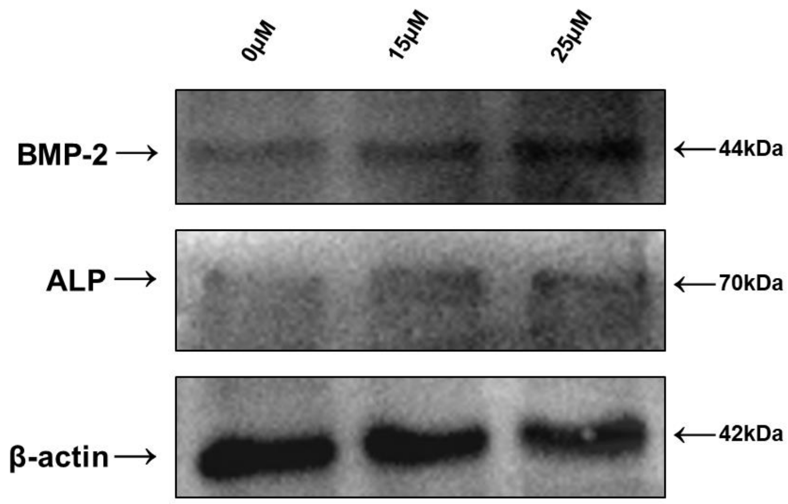

Relative expression of marker genes during osteoblast differentiation such as ALP and BMP-2 at protein level.

Supplementary Figure S15: **Compound 17 restrains the release of CTX-1 after treatment with compound 17 during inflammatory osteolysis *in vivo*.**

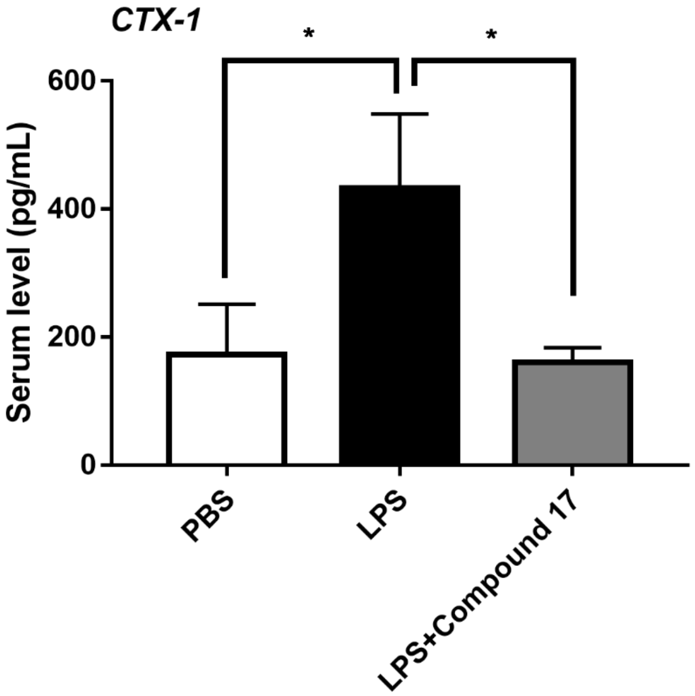

Serum CTX-1 level was detected using a mouse CTX-1 ELISA kit (Cloud Clone CORP, Wuhan, China) according to the manufacturer's protocol.
